# Quantitative Agent Based Model of Opinion Dynamics: Polish Elections of 2015

**DOI:** 10.1371/journal.pone.0155098

**Published:** 2016-05-12

**Authors:** Pawel Sobkowicz

**Affiliations:** KEN 94/140, Warsaw, Poland; Université Toulouse 1 Capitole, FRANCE

## Abstract

We present results of an abstract, agent based model of opinion dynamics simulations based on the emotion/information/opinion (E/I/O) approach, applied to a strongly polarized society, corresponding to the Polish political scene between 2005 and 2015. Under certain conditions the model leads to metastable coexistence of two subcommunities of comparable size (supporting the corresponding opinions)—which corresponds to the bipartisan split found in Poland. Spurred by the recent breakdown of this political duopoly, which occurred in 2015, we present a model extension that describes both the long term coexistence of the two opposing opinions and a rapid, transitory change due to the appearance of a third party alternative. We provide quantitative comparison of the model with the results of polls and elections in Poland, testing the assumptions related to the modeled processes and the parameters used in the simulations. It is shown, that when the propaganda messages of the two incumbent parties differ in emotional tone, the political status quo may be unstable. The asymmetry of the emotions within the support bases of the two parties allows one of them to be ‘invaded’ by a newcomer third party very quickly, while the second remains immune to such invasion.

## 1 Introduction

The changes of collective political opinions resulting from individual interactions between people and from external influences of persuasion efforts of various interested parties and organizations are part of the core of topics of sociology. More than 150 years ago, Auguste Comte [1] imagined social physics as *that science which occupies itself with social phenomena, considered in the same light as astronomical, physical, chemical, and physiological phenomena, that is to say as being subject to natural and invariable laws of discovery of which is the special object of its researches* (as described by Iggers [2]). Still, the dream of achieving in the description of social phenomena the predictive accuracy typical for the physical sciences has been unattainable. The reasons come from many facts, not least of them being the complexity of our societies and lack of empirical, quantitative data on both the individual and group behaviors. As Castellano, Fortunato and Loreto note in their review of the developments of the use of statistical physics in the studies of social phenomena [3], *it is only in the past few years that the idea of approaching society within the framework of statistical physics has transformed from a philosophical declaration of principles to a concrete research effort involving a critical mass of physicists. The availability of new large databases as well as the appearance of brand new social phenomena (mostly related to the Internet) and the somewhat specular tendency of social scientists, that are moving toward the formulation of simplified models and their quantitative analysis, have been instrumental for this change*.

The interdisciplinary field has acquired a nickname of *sociophysics*, and it is not surprising that the studies of opinion changes in societies are one if its main interests. One of the reasons is the importance of understanding of changes in public attitudes versus specific issues or policies. The second is that, at least in the initial stages, the qualitative similarity between the sudden opinion swings and magnetization phenomena allowed to use a ready-made mathematical apparatus. There are many approaches to the topic, differing in the description of the available opinion states, interpersonal dynamics, social network description and many others. Among the most popular, one can mention the voter model [4–7], the Sznajd model [8–15], the bounded confidence model [16–21], the Hegelsmann-Krause model [22], the social impact model of Nowak-Latané [23, 24] and its further modifications [25–28].

These sociophysical models have resulted in considerable advance in the understanding of some common, universal mechanisms of social opinion change, using statistical treatment of the interactions between large numbers of people. Examples of such discussions may be found in [29, 30]. In addition to introducing descriptive measures of chaotic behavior or quick social changes, derived from physics (entropy, phase transitions), the sociophysical works have provided several qualitative analyses of social systems.

So far, however, the quantitative description of an opinion change in a real social system has been quite elusive [31]. Only a few works have attempted such a task with respect to election results and voter dynamics. For example Caruso and Castorina [32], by restricting their models to bipolar party systems, were able to describe the elections in Germany and Italy, and to offer some limited predictions. Fortunato and Castellano [33] have discovered and described the phenomenological universality in the lognormal distribution of of the number of votes received by candidates, identical in different countries and years. This universal distribution was found to be reproduced by a simple dynamical model for the behavior of voters, similar to a branching process. This year brought two more works [34, 35], devoted, respectively, to the Portuguese and Brazilian elections.

In addition to the studies of group behavior, there are a few attempts at achieving a quantitative agreement between observations and agent based models in the domain of describing a single voter. A rather complex model presented by Kim, Taber and Lodge [36] allows to describe, with quite good agreement with reality, the campaign responses of American voters, depending on their Liberal/Conservative leanings. The agent based model reproduces responsiveness, persistence, and polarization of political attitudes, as well as the responses to Liberal/Conservative media. The quality of the simulations strongly supports the idea of the centrality of motivated reasoning to the processes of candidate evaluation.

The goal of our current work was to provide a quantitative description of the medium term (ten years) evolution of the party preferences in Poland. It is based on a modified version of a previously published model of social opinion change, which takes into account the interplay of emotions and information in the individual opinion change processes [37–39]. The model is used to describe quantitatively the rapid changes on the Polish political scene in 2015. In fact, the model has allowed a quantitative prediction of the evolution of the support for the main parties ahead of the parliamentary elections held in October 2015.

Since the overthrow of the communism, Poland has experienced a high rate of the evolution of the political system. It is coupled with a rapidly growing level of political polarization, that includes media to a higher degree than the US. For these reasons, Poland provides an important testing ground for the theories of the voting behavior and media influences that may be applicable to other countries.

Our goal is to present a model that would be simple enough to understand intuitively, yet which would be based more closely on psychological understanding of human behavior than the standard sociophysical approaches. The approach is based on an abstract, agent based simulations, in which the global system behavior—such as the support for the political parties—results from combined interactions of relatively simple agents. The internal dynamics of the agents takes into account some of the complexity in the individual human behavior, by using some concepts of the catastrophe theory of behavior [40]. The model includes also the influence of the media on the large scale preferences of the electorate. The model parameters are probabilities of certain reactions to a given type of a social contact or news. They were chosen to obtain the best agreement with the recorded party support data, but they include no specific reference to the actual political programs, persons or events. In addition to reproducing quantitatively the evolution of the support for the political parties (as measured by poll results and elections), the model shows a qualitative similarity with the evolution of support on an individual constituency levels.

We note here, that the predictive capacity of the model is, in large part, due to a relatively simple situation in Poland prior to 2015. This has allowed an effective estimation of the model parameters—a task that is the most difficult in the process of connecting the real societies to the abstract models. Such simplicity of the initial conditions is most likely absent in other countries and political systems. Still, to our knowledge, the present work is rather unique in the capacity of modeling a complex, real society.

The paper is organized as follows. W start by introducing shortly the Polish political situation. This seems necessary to understand the basic premise of the asymmetry in the political propaganda, present in Poland but largely absent in other highly polarized societies (such as the United States). We the proceed to describe the model and the modifications particular to the current work. This is followed by the results of the model and the discussion of the dependence on the parameters. The last section provides a discussion of the results and some considerations related to possible further applications of the model.

## 2 Political situation in Poland

### 2.1 Bipartizan stalemate

**Fig 1:**
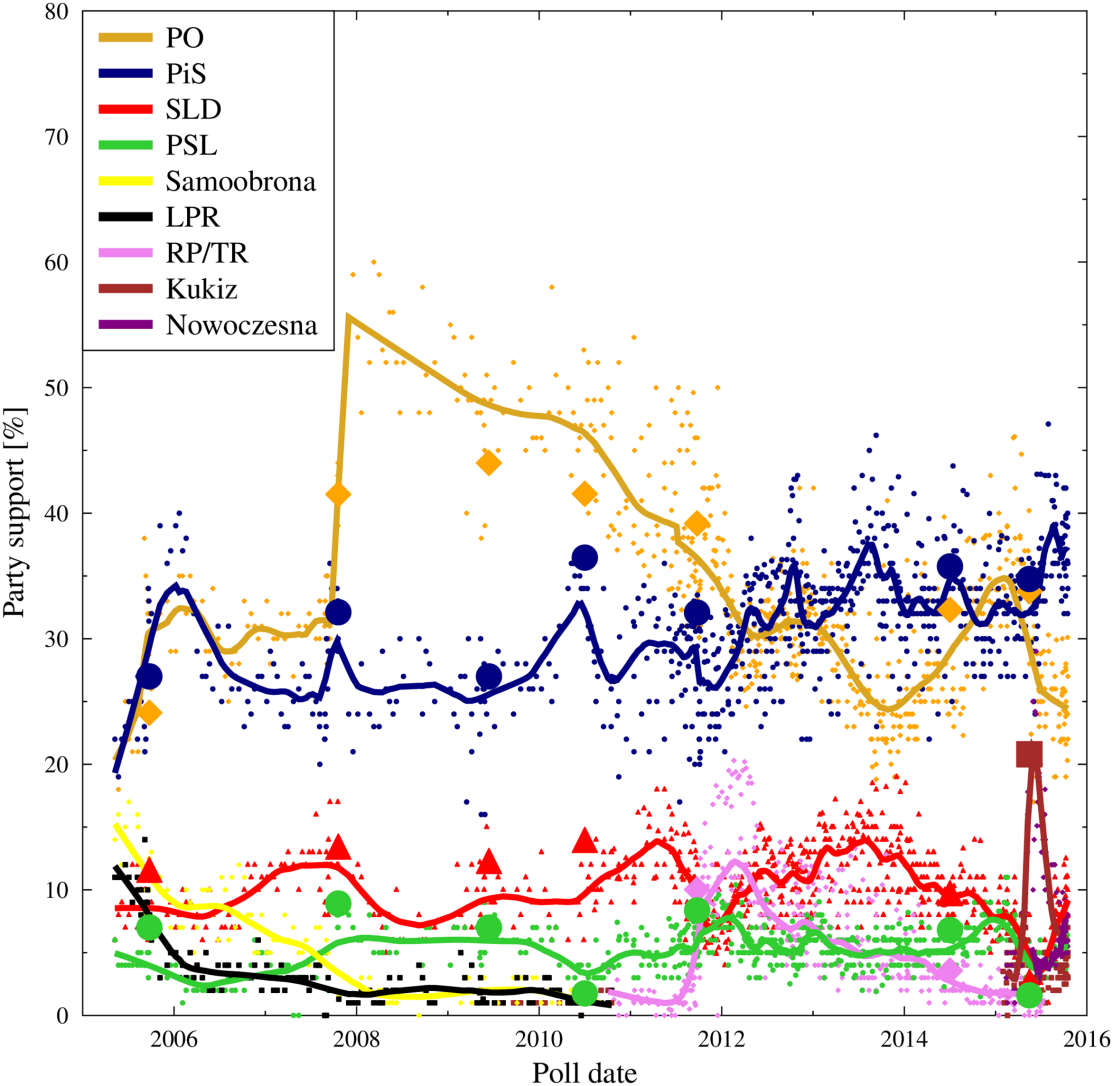
Evolution of the support for major political parties in Poland 2005–2015. PO and PiS are the main contenders, while SLD (socialdemocrats) and PSL (peasants party) are smaller ones, existing on the scene during the whole 25 year post-communist era, with small but well entrenched core electoral base. The data on PiS group them together with two other parties, PJN and SP, which have split-off from PiS during the period, but which have since returned to form a single political entity in late 2014. As the three parties address the same electorate with very similar propositions, we treat them together in the polls analysis. Since 2007, PSL is the coalition partner of PO. Samoobrona and LPR were the coalition partners of PiS during 2005–2007, and essentially vanished from the political scene after the 2007 elections. The jump in the popularity of PO after the success in the 2007 elections is, most likely, an emotional reaction to the defeat of PiS and the end of the so called ‘4th Republic’. RP/TR (Ruch Palikota/Twoj Ruch) is a party formed in mid-2011 by a PO dissident, which has enjoyed a brief period of success between 2011 and 2012. Large symbols denote the results of the elections (parliamentary, Europarliament and first rounds of the presidential elections). Kukiz and Nowoczesna are new entrants in 2015. Data collected from various sources, with the main contribution from Mr Maciej Witkowiak, http://niepewnesondaze:blogspot:com/ and the WEB platform http://ewybory.eu/.

During the 25 years since the overthrow of the communist rule, Poland has had a quite active political history. Fig 1 shows the evolution of support for several main parties during the last ten years, 2005–2015. With a single exception in 2011, every parliamentary election brought a radical change in the landscape of political parties and the ruling coalition. Since 1989, the post-communist election rules were changed profoundly many times: initially they allowed people to vote for candidates representing multiple parties; later they changed to votes for one party, in a proportional system with rather high threshold of 5%. This later change was coupled with a growing polarization of the political scene (and the whole society), which has led around 2005 to a dominance of two parties: Platforma Obywatelska (PO, Civic Platform) and Prawo i Sprawiedliwość (PiS, Law and Justice). This is the period we are focusing on in our analyses. The description presented below corresponds to the view of the Author, for additional, peer reviewed information we suggest to consult, for example [41–50].

It is rather interesting, that in the parliamentary elections in 2005 which brought the two parties to the dominant positions at the expense of the leftist parties, the election campaign was based on the promise of a joint government, with the unofficial, but popular, acronym POPiS. This was considered by many voters to be a very attractive political idea, and the two parties got 28.91% and 33.70% of the votes, respectively. No other party received more than 12% of the votes. The support for the previous winner SLD (social-democrats) dropped from 41.04% (in 2001) to 11.31% in 2005. Despite the pre-election declarations of coalition, shortly after the success which has brought the two parties to the forefront of the Polish political scene, a conflict between the leaders of the two parties broke out. The promised coalition never happened, leaving a part of the electorate dubbed ‘POPiS orphans’ deeply unsatisfied (and somewhat divided as to whose fault it was). The government was formed by PiS, led by Mr Jarosław Kaczyński, in coalition with two smaller parties, Samoobrona and LPR. This proved untenable, and in early elections in 2007 due to the crisis, it was PO that captured the majority (41.51%), while PiS has became a strong opposition, with 32.11% of votes. This result was nearly repeated in the next parliamentary elections in 2011, which was won by PO (39.18%) with PiS receiving 29.89% of the votes.

Since 2005 until 2014, the two parties have enjoyed a virtual dominance on the Polish political scene, as documented not only by the results of the parliamentary elections but also the presidential ones, elections to the European Parliament and local elections. It is also confirmed by the data from popularity polls, which show practically stable, high level support for the two dominant parties with a stable or decreasing popularity of the minor parties, which have rarely passed over the 10% popularity barrier. The stability is even more visible if one adds to the results of PiS the figures for two smaller parties: PJN and SP, which have split off from PiS in 2011 and 2012, due to personal differences, keeping a very similar political program and addressing the same electoral base. The two parties merged back with PiS in 2014. In all further considerations we shall consider the joint PiS/PJN/SP data.

The jump in the popularity of PO in the aftermath of the 2007 elections—from about 30% to about 50% is a phenomenon deserving a deep explanation, which is, however, beyond the scope of this work. Most likely, it is connected with an emotional effect of defeating PiS. Between 2007 and 2012, PO had a clear, but slowly diminishing lead over PiS, seen not only in the polls but also in the results of the elections. Since 2012, the leading position became more contested, with periods in which one or the other party enjoyed a better position, each oscillating around 30% of the support (Fig 1).

**Fig 2:**
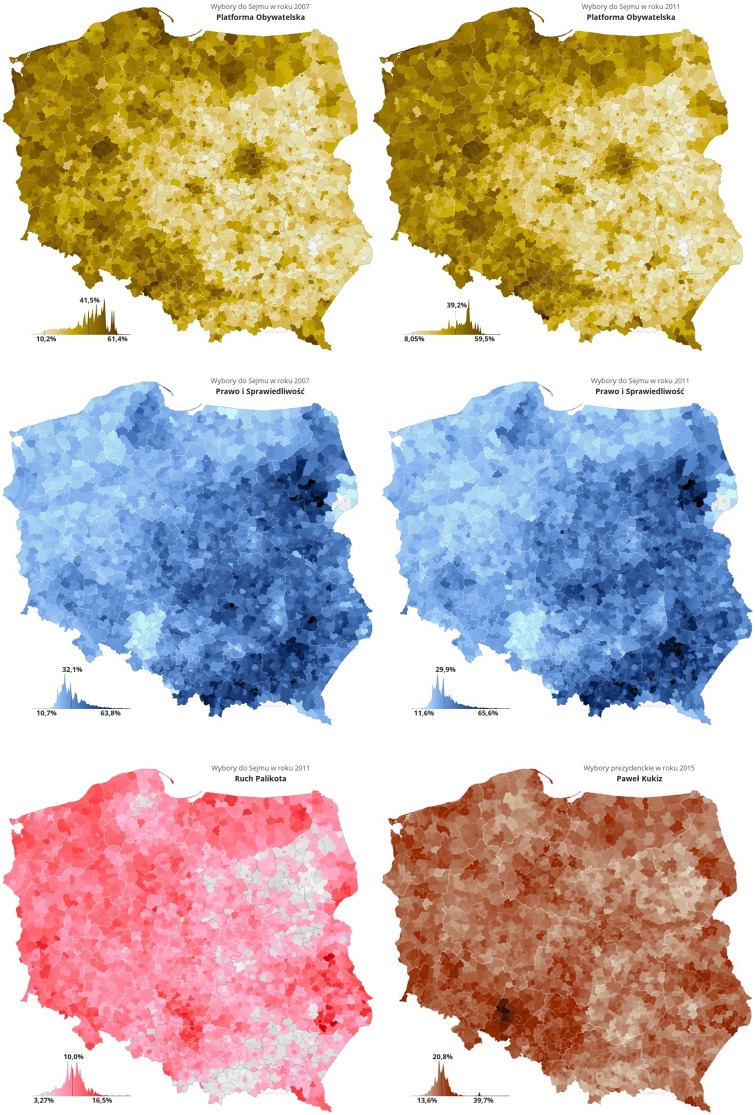
Evolution of the local community voter preferences in Poland. Top row: PO support in parliamentary elections in 2007 and 2011, middle row—corresponding support for PiS. There is a clearly visible, stable geographical division of the support for the two parties. Bottom row shows the results achieved by the two ‘challenger’ parties: left panel: results of RP/TR (Ruch Palikota/Twoj Ruch) in 2011, corresponding to the peak in its popularity—most of the votes come from the North-Western Poland constituencies, where PO dominates (with the exception of the Lublin region in southeast Poland, the home town of Mr Palikot, the party leader). Similar correlation is visible in the votes for Mr Kukiz in the presidential elections of 2015 (bottom row, right panel). Here too, the highest support occurs in the constituencies where previously PO was dominant, and is much weaker in the PiS dominated regions. Data and maps courtesy of Jan Borkowski http://www.mapki.ga/.

There are multiple explanations for the duopoly that emerged in Poland in 2005, offered by the political commentators. On one level, it is quite illuminating to consider the map of voter preferences, which shows remarkably stable pattern of support, roughly corresponding to historical boundaries of Polish partitions in 19th century. Such geographical division of preferences corresponds to deeper cultural patterns, which will not be discussed in this work. We note that the changes in the voting results take the form of gradual shifts of the dominance in the constituencies, as shown in Fig 2.

Another reason for the stability, acting in parallel to the socio-geographic one, is the capture of the public debate by PO and PiS, based on emotional appeals of the two conflicted parties. In our opinion this is the crucial factor in the creation of long term bipolar societies. Ever since the elections of 2005, the would-be coalition partners started an aggressive fight, leading to a very strong polarization of the Polish population and media. Much of the attention was due to the highly emotional personal differences between the leaders of the two parties: Mr Jarosław Kaczyński (PiS) and Mr Donald Tusk (PO). The conflict has left very little room in the public debates for any other political party—phenomenon known in other countries (e.g. the dominance of the Democrat/Republican conflict on the US political scene). The public campaigns of both parties focused on negative emotions, accusations, fear of what would happen if ‘they’ win, etc. This aggressiveness has increased, especially on the part of PiS, since the crash of the plane carrying the Polish President, Mr Lech Kaczyński (twin brother of the PiS leader) near Smolensk in Russia in April 2010. In the subsequent presidential elections of 2010, Mr Jarosław Kaczyński (who stepped in to continue his brother legacy) lost to the PO candidate, Mr Bronisław Komorowski. In this way PO has achieved full control of the government structures: parliament majority with a single coalition partner, government and the presidential office (but short of of reaching the capacity to change the constitution). The importance of the personal loss for Mr Kaczyński were tremendous, as his links with the twin brother were very strong.

The tragic plane crash and the lost elections have significantly increased the negative tone of the PiS communications with the electorate. In addition to the typical (for a political opposition) criticism of mis-management of the country, a significant part of the message focused on accusations that the plane crash was a result of some sort of a conspiracy, and that the plane was destroyed by a mid-air explosion, variously attributed to a rocket or a bomb planted on board. Implicitly, the PiS propaganda indicated Russia as the culprit, but the direct target was the PO government, either as acting in collusion with the perpetrators of the attack, or, in the least aggressive case, as grossly incompetent in handling of the crash investigation and actively covering the wrongdoers.

The increasing conflict and the personal attacks resulted in an increasingly polarized society. Moreover, the polarization covers also the media outlets: there are TV stations, daily journals, weeklies and WEB portals that cover almost exclusively a single viewpoint. There is strong tendency to limit the range of the media used by the supporters of each political camp to the outlets representing the same views, known as selective exposure [51–55]. The opposing views and news media are categorized as traitorous or trash, and largely disregarded or, even more, serve to strengthen ones own views via motivated reasoning [56–60].

The aggressively negative communications by PiS is in stark contrast with the communication strategy chosen by PO. For example, the government has been very late in mounting an information campaign countering the conspiracy theories of the Smolensk crash embraced by PiS. Relying on the results of the official investigations, the messages were long delayed, and lacked the emotional appeal. At the same time, shortly after the presidential elections in 2010, Mr Tusk declared that ‘as long as he is active in public life, he prefers, what some mischievous commentators call warm water in the tap policy’ (Source: Gazeta Wyborcza, 20 Sept 2010, http://wyborcza.pl/1,76842,8397290,Tusk__Wole_polityke_cieplej_wody_w_kranie.html). This sentence has became a byword in the political discussions since then. In fact, most of the communication strategy of PO focused on the achievements of the government and country as a whole, its economic growth, new infrastructure such as highways, etc. It should be noted here, that during the period in which PO was in power, Poland has indeed enjoyed significant benefits of the EU membership and has weathered the economic crisis with remarkable success, being the only country to preserve continuous growth of GDP (dubbed ‘green island’ by the PO government). Thus the ‘success’ focused communications did have a real background. But the message did not evoke emotional interest and commitment on the part of the PO supporters comparable to the effects of the negative campaign of PiS.

The local elections held in November 2014 resulted in a tie between PO and PiS, with a very strong result for the PO coalition partner PSL. Again, the way these results were presented in the respective party communications was very different. The government camp has declared yet another victory in a long string of dominance. PiS, on the other hand, has openly claimed that the elections were manipulated and the results rigged. It is no surprise that the emotional response in the aftermath of the elections was very different as well: while the PO voters mostly did not take much notice (quite natural with the tone of the party propaganda being ‘business as usual’), the PiS electorate was actively mobilized and formed many ‘spontaneous’ initiatives ‘to protect future elections’.

### 2.2 Breakdown of the duopoly

**Fig 3:**
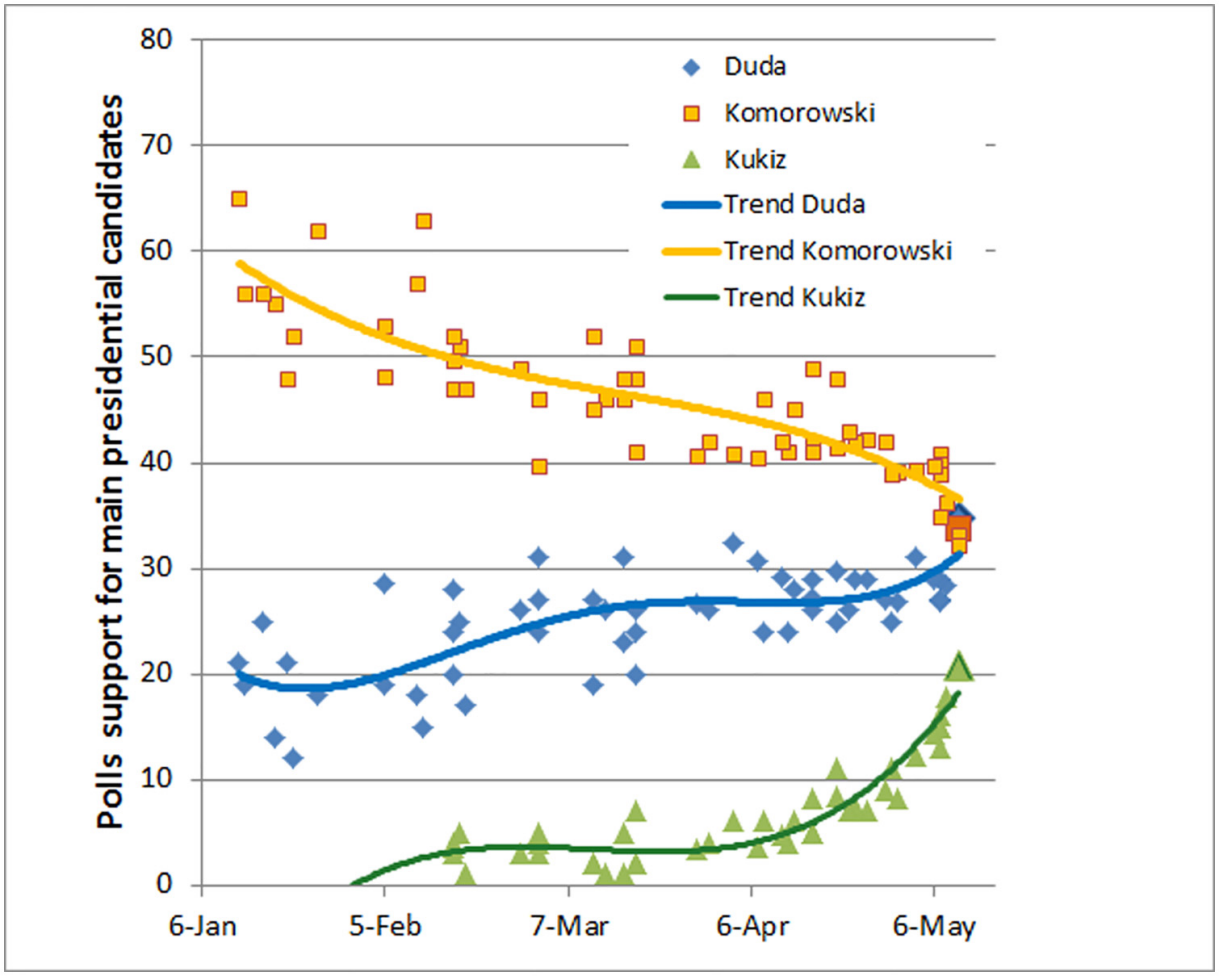
Evolution of the support for the candidates in the presidential elections of 2015. Mr Komorowski (PO) and Mr Duda (PiS) have entered the race in late 2014. The figure includes the data on the support for the independent candidate, Mr Kukiz, who has appeared in the polls in late February. The final (larger) points indicate the results of the first round of voting in the presidential elections (Md Duda: 34.76% of the votes; Mr Komorowski: 33.77%; Mr Kukiz; 20.8%). Data from various polls, via Wikipedia.

The long period of relative stability of the PO-PiS duopoly described above broke down during the presidential elections held in May-June 2015 (Fig 3). The early polls, in January 2015, have indicated a landslide victory of the incumbent president, Mr Komorowski, with over 60% support, allowing him to win in the first round of voting (Fig 3). The expectation of an easy win was further enhanced by the choice of a candidate by PiS (instead of the party leader, Mr Jarosław Kaczyński, the party has chosen as its candidate Mr Andrzej Duda, almost unknown to the general public). This was generally commented as avoidance, on the part of PiS, of another loss by Mr Kaczyński to a PO candidate. However, lulled by the poll results, the incumbent has led a lackluster (some commentators said ‘nonexistent’) campaign, expecting an easy victory. This resulted in a steady loss of support. At the same time, the poll results for Mr Duda have grown somewhat, from below the party support to the values roughly equivalent to it. It should be noted here, that the campaign of Mr Duda was much more toned down and focused on his image as a rational, future oriented politician, skirting the typical PiS aggressive messages. Eventually Mr Duda narrowly won the first round (gathering 34.76% of the votes, compared to 33.77% for Mr Komorowski) and the second round (although by a rather narrow margin 51.55/48.45%). We stress here that the closeness of the vote should be compared to the original, almost 40% lead of Mr Komorowski.

The phenomenon which has prompted our current analysis, was an appearance, seemingly out of nowhere, of a third significant candidate and the related breakdown of the political duopoly. An independent candidate, Mr Paweł Kukiz (an active rockman, who previously has not been much active in politics) started his campaign almost two months after the main rivals’, without significant funding, but has quickly became an anti-establishment symbol and has focused the attention of the dissatisfied, especially the young part of the population. Based on a battle cry of single-member constituencies as a solution for the fall of democracy in Poland, (instead of the proportional system, a change which would require a constitutional majority), Mr Kukiz appealed to all the disenchanted with the current party system. In the space of a few months he has gathered an enormous support, eventually getting 20.8% of the votes in the first round of the elections (see Fig 3). It is worth noting that no other candidate got more than 3.5% of the votes, with the majority receiving less than one percent. Thus the three candidates—two of them representing the dominant parties and one, coming completely from outside—have really stood out in the popular view.

The first research question is therefore: where did the popularity of Mr Kukiz come from, and in what way his campaign influenced the losses of PO candidate and the gains of PiS one? The second question is: could the model tell anything about the future developments?

The second issue was important because of the parliamentary elections scheduled for the late October, that is five months after the presidential ones. To understand this period we note that the electoral strategies of the two largest parties between June and October 2015 were fairly predictable.

PO, dismayed by the loss of the presidency, despite the initial advantage, has attempted to focus on messages beyond the ‘warm water in the tap’, but without losing the image of calm and rational party. This was only partially successful, as there were no significant, emotionally mobilizing topics other than a possible strategy of exploiting the fear of the ‘return of PiS’. Moreover, in the absence of Mr Tusk, the leadership of his successor, Ms Ewa Kopacz, had much less decisive edge.

PiS, on the other hand, emboldened by the success of the strategy of ‘hiding’ the highly controversial persona of Mr Kaczyński, has decided to repeat the model for the parliamentary elections. This time, PiS has chosen again a relatively less known Ms Beata Szydło as the face of the campaign and candidate for the Prime Minister post. This has provided a symmetry to Ms Kopacz. The tone of the PiS propaganda, as in the case of the presidential elections, was much calmer than previously, especially when addressed outside of the core support base. Only in late September and October, did Mr Kaczyński and Mr Maciarewicz (another highly controversial PiS member) make public statements. In fact, some commentators attributed a drop in the poll results of PiS to these appearances.

The difference between the strategies of PO and PiS and Kukiz’15 is also clearly visible in respect to the European refugee/migration crisis. The official policies of the PO led government were (rather cautiously and in a limited way) supportive of the EU policies of allowing the refugees within the union countries. The media outlets connected with PO focused on the humanitarian aspects and denounced any negative associations related to the massive migration. On the other hand, the opposition has argued against any acceptance of the refugee quota, and stressed the need for independent, national policies. Here also, the viewpoint of the associated media was more extreme, building the fears and negative emotions via coverage of the incidents involving the migrants. The difference in the tone of the media related to the refugee crisis translated directly into a strongly motivational effect based on fear (for the PiS and Kukiz supporters) contrasted with only slightly motivational one, based on ethical principles combined with demotivation based on the government decisions taken without consultations (for the PO supporters).

The lack of Mr Kukiz experience in the ‘professional’ politics was clearly visible in his actions after the presidential elections. Almost immediately after the first round, Mr Kukiz has declared the intention to form a political movement and to prepare for participation in the parliamentary elections in October. The communication strategy chosen by Mr Kukiz after his success was, however not too successful. Instead of building up on the foundation of his personal success and the image of a game-changer, he has begun to quarrel with his staff almost immediately after the presidential elections. This resulted in a difficulty in the creation of a country-wide representation, needed for the parliamentary push. When, finally, the Kukiz’15 committee lists were assembled, they included many candidates with radically opposing views, united only by their anti-establishment rhetoric. As a result, the appeal of the Kukiz team was much weaker than his personal one in the case of the run for the presidency. The capacity of Kukiz’15 to increase and sustain support dropped significantly, which was promptly reflected by a drop in the poll results. Moreover, this drop resulted in a loss of the interest of the media in Mr Kukiz, which drove the decrease even further.

**Fig 4:**
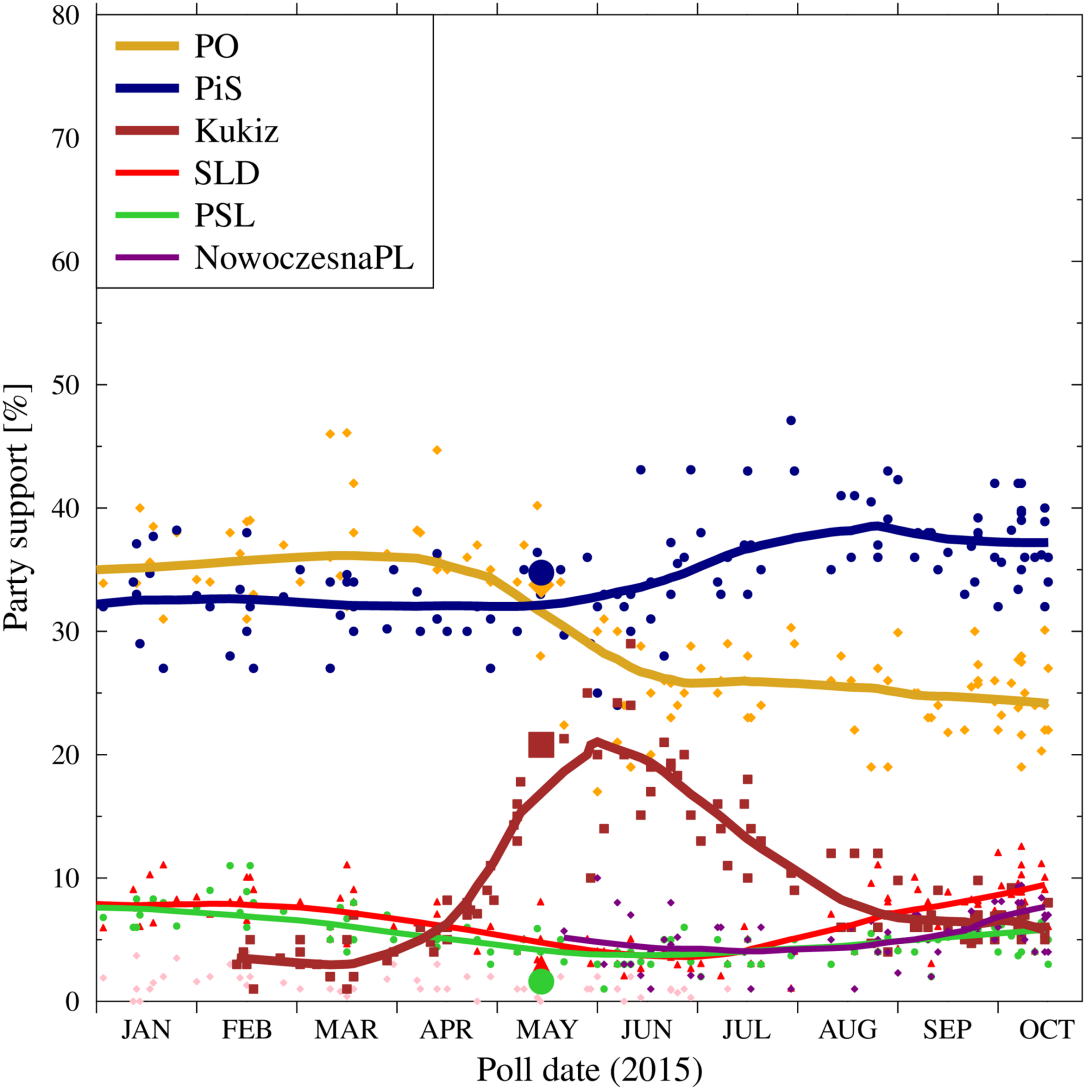
Evolution of the support for major political parties in 2015. We draw the attention to apparently flat support for PiS and PO since July, when the results of the presidential elections set in and the parties were fully involved in the parliamentary elections. Suppoprt for Mr Kukiz in the presidential elections is spliced with thew support for the Kukiz’15 committee in the parliamentary ones. Data collected from various sources, with the main contribution from Mr Maciej Witkowiak, http://niepewnesondaze:blogspot:com/ and the WEB platform http://ewybory.eu/.

As shown in Fig 4, since July, when the parliamentary campaign was in full swing, the support for PO and PiS, as measured by the polls, remained relatively steady. At the same time, the gains of Mr Kukiz from the March-May timeframe evaporated quickly. Part of the loss was compensated by the increase of support for the smaller parties: SLD, PSL,. Nowoczesna and KORWiN, however all of these remained below 10%. The situation continued until October.

Due to the quite large values of the election thresholds in Poland (5% for parties, 8% for the declared coalitions) even just before the elections, the actual parliamentary composition has remained uncertain. Only PO and PiS were certain of their place in the future parliament, while all other parties were close to the thresholds. In fact, in the five polls conducted on October 22nd, the parliamentary composition varied between three parties and seven.

## 3 General model description

The observation of the rapid change of the political situation after the long period of quasi stability has motivated us to attempt a construction of a sociophysical model that would describe it. While stable political duopolies have already been studied via agent based models, the sudden breakdown of such situation provides an interesting theoretical challenge. In our work we aim to formulate a single conceptual frame, covering the existence of the meta-stable duopoly and its recent breakdown. Both are explained via the individual opinion dynamics that stresses the importance of the emotional influences. We focus on the dominating effects of the asymmetry of the propaganda strategies of the two main parties leading to differing susceptibility of the support base to external influences.

Part of the model legacy comes from extended studies of the behavior of the users of Internet fora related to the Polish politics between 2009 and 2011, where we have observed strong correlations between the expressed opinions and emotions of the participants [61–63]. In particular, we have observed that when the emotional arousal of the participants is high, their capacity to change opinions is negligibly small. This observation has led us to propose an Agent Based Model in which the individual opinion about a specific issue would be influenced by a combination of the information related to the matter and the emotional state of the agent. Due to this combination, we refer to the dynamics of the opinions as the Emotion/Information/Opinion model (E/I/O for short). The model was introduced and described in considerable detail in [37] and [38]. Its structure combines ‘microscopic’ description of individual opinion change with a flexible communication mechanism, allowing to use the model in different social contexts. Most of the parameters describing the interactions between the agents in the current work are taken from these earlier studies.

### 3.1 Individual interactions

The proposed solution is based on a simplification of the catastrophe theory of behavior, which has been introduced more than 30 years ago [40], and had been used (and criticized) in analyses of human behavior (for a review see [64]).

In modeling of attitude change the most popular catastrophe theory approach was based on the cusp catastrophe, which allowed to intuitively explain a hysteresis behavior [65–73]. The person’s behavior is assumed to depend on two continuous variables generally named the normal factor and the splitting factor. For low values of the splitting factor, the normal factor (such as the information about the issue in question) completely determines the outcome. However, the increase of the splitting factor leads to appearance of a region where the agent may have one of the two opposing opinions for the same value of the available information. The splitting factor has been variously identified as the emotional involvement in the issue, the strength of the individual preconceptions or the importance of the issue for the individual. Still, even with a number of variables limited to two, because of the continuous nature of the parameters, the application of the catastrophe theory to real life is extremely cumbersome, as shown, for example by [68, 74–79]. The difficulties arise from the need to precisely measure and assign numerical values to the control variables, which are often of a delicate, psychological nature: information, involvement in the issue, personal importance, emotional factors, and the difficulty of assigning numerical values with high precision.

If obtaining the values of the continuous cusp catastrophe parameters from observations in attempt to understand specific social examples is difficult and prone to large errors, then the effective use of agent based models for predictive purposes, using the same framework, is even more difficult. We must remember that the quality of any simulation in reproducing real world data crucially depends on the ability to determine the proper input parameters, and the more errors are inherent in this choice, the more difficult is to check if ‘this is the right combination of parameters’, corresponding to the studied social system.

In this situation we propose a simplified discrete model that includes the key property of the cusp catastrophe, that is the possibility that for certain emotional states it is possible for the agents to hold conflicting opinions despite the fact that they share the same information about the issue (corresponding to the fold in the cusp catastrophe surface). This also allows the ‘hysteresis’ behavior when the normal factor is changed continuously back and forth. The discrete states describing the agent characteristics correspond to idealized, crucial states on the cusp catastrophe surface. For graphical visualization of the discretization approach we refer the Reader to Fig A in S1 File.

Regarding the use of emotions and information as the two *independent* variables, we note that such dichotomy has a long tradition in the psychological literature. The scientific verdict is still out on whether the emotions are primary to the cognitive processing of information, as Zajonc postulates [80, 81], or, as in the opposing view of Lazarus [82], they may result from the cognitive functions. In fact, as the review of Petty and Wegener [83] shows, the psychological literature on persuasion and opinion change includes many conflicting theories which consider tens of distinct variables influencing the process. Part of this conundrum is possibly related to differences in meaning, but it is reasonable to assume, that the path linking various (often co-existing) emotions and cognitive functions and expressed opinions and associated actions is truly quite complex. In such light, the focus on just two parameters ‘driving’ the opinion change: the available information and the emotional state is an obvious simplification. On the other hand, such simplification, used in a working model, may lead to potentially useful understanding of the processes—one of the major goals of the model based approach [84, 85]. In terms it role as the splitting factor, the emotional state is less dependent on the valence of the emotions (positive or negative) than on their intensity. We note also, that it seems to be much easier to evoke, on a massive scale, strong negative emotions, such as fear, anxiety, hatred or disgust. For these reasons, in the E/I/O model we treat the processing of information and the emotional influences on an equal footing, with a nonlinear interplay leading to the final results.

While the catastrophe theory has been used in description of the individual behavior, it is not well suited for large scale opinion simulations. This is because the continuous nature of the control variables makes it very difficult to correctly map the model and psychological observations and then to assign these values to computer based agent societies—there is simply too much variability in the starting conditions and system evolution.

For this reason we have proposed [37, 38] a discrete version of the approach, in which the continuous folded cusp surface is replaced by just seven states corresponding to two values of the emotion level (splitting factor): calm and agitated and three values for the information and opinion: pro, contra and neutral. The transitions between the agent states in the discretized version of the cusp catastrophe may be described more efficiently, and there is less ambiguity in determining the agent’s behavior.

We note here that instead of using the full, multifaceted spectrum of possible emotions, or even a positive/negative valence measure of emotions, we focus on their intensity. Moreover, im most cases the intensity is associated with the negative emotions, as these play a more influential role in shaping the motivated information processing [86–89].

It should be noted that the catastrophe theory, even without the simplification that condensed the complex, two-dimensional folded plane into a finite set of discrete states, does not account for several features important in the real world opinion dynamics. For example, it does not cover the effects of trust, reputation and charisma. There is no place for any individual character traits, such as the tendency for a contrarian behavior or anticonfirmism [90, 91]. Nevertheless, even the simple act of combining the multitude of the variables potentially influencing the opinions into the two groups: the driving factor (information about the issue, trust in its source, its reputation etc.) and the splitting factor (involvement in the issue, selfconfidence, commitment, emotional state) can enrich the operational treatment sufficiently to allow descriptions of complex social situations [65, 74, 92–94].

We are using an approach based on communication between via separate messages, rather than a continuous interactions. This allows to describe the time flow efficiently. Any message would be described by the same set of variables as the agents themselves: emotional arousal level, information and opinion—corresponding to the source of the message. In the case of messages sent by an agent, the characteristics of the message are assumed to equal those of the authoring agent.

Upon receiving a message (which means either interacting with another agent or with media carrying some propaganda messages), the recipient state may, in some cases, be modified. Following the ideology of the catastrophe model, we assume that agents in calm states are capable of changing their opinion, if they receive information contrary to their current beliefs (calm state allows rational processing of such information leading to change of the opinion). On the other hand, agitated agents, when exposed to contrary information, would preserve their opinion unchanged, refusing to process and accept the messages in a rational way. Thus, within the model, the only way to make an agitated agent change its opinion is first to calm down (which may happen with probability *p*_*calm*_, set at 20%, if the agent is in contact with calm messages or agents with which it is in agreement), and then by changing the opinion by contact with messages or agents with an opposing view. In addition to the ‘calming’ processes, a reverse process in which a calm agent exposed to contrary view may become agitated (without changing the opinion). This happens with a probability *p*_*agit*_, set at 20%, when the contact is with a calm opponent/message and with a 100% probability if the opponent is agitated. The details of the effects of the interactions with other agents are described in Table A in S2 File.

These basic rules correspond to simplified, intuitive psychological description of opinion change of a person, dependent on the current opinion and emotional state of that person, as well as on the state of the person with whom the interaction occurs, again, both the opinion and the emotions.

### 3.2 Treatment of propaganda effects

As already mentioned in the current scenario we consider both direct interactions between pairs of agents and agent’s reactions to global messages, corresponding to the media coverage of the political issues and party persuasion campaigns. The latter are generated within the with specific opinions and end emotions (corresponding to the propaganda strategy of the political parties), issued with pre-fixed frequency.

In the description of the effects of the messages transmitted by the media, we used a simplification of assuming that the stream of the messages is divided into separate sub-streams representing the communication efforts of each of the parties. There are two justifications for such simplification. The first is the phenomenon of selective exposure. The second is due to the extremely high level of polarization of the Polish media [95–97]. The parties usually find ways to effectively plan and execute their media strategies, using their ‘friendly’ media. Some information channels (newspapers, weeklies, radio/TV stations, WEB sites) serve directly as outlets of almost unmodified persuasive messages, provided they come from the ‘right’ party public relations department. Others have clearly defined preferences and biases, even if they ‘package’ the content in a less blatant way. Only a minority of the media important for the political arena have retained some degree of impartiality and neutrality.

For these reasons, within our analysis we have equated the news and comments stream influencing the population with party propaganda. While this simplification may be unjust for some journalists, editors, channel managers, it is, in our opinion, reasonably justified by the current Polish media market situation.

We note here that in our opinion the external sources (media, propaganda) play a more important role in shaping the opinions and emotions related to the political issues in Poland than the personal contacts. It is quite common for people to seek contact with other people who share their opinions and emotions. In social environments where a free choice of companions is not possible, for example in the workplace and family relationships, people tend to avoid the sensitive topics, in order to minimize the conflicts. These tendencies minimize, to some extent, the hostility generated in the person-to-person contacts.

On the other hand, the media thrive on the polarized messages, give them the front page coverage, highly memorizable graphic context and often exaggerated content. For these reasons we have assumed in the simulations that for each agent, on average, 80% of the political messages come from party propaganda sources, while only 20% are result of interactions with social neighbors. The 20% of the messages between the agents are generated directly from the internal states of the agents: their emotions and opinions. As shown in [38], for short range agent-to-agent interactions (as assumed here), the effect of these direct interactions is to create local communities sharing the opinion, which may be remarkably stable, due to the localized ‘echo chamber’ effect, in which most interactions are with like-minded agents.

The remaining 80% of messages, attributed to propaganda, is divided between the competing parties, and within these divisions, into the four categories: internal mobilizing, internal demobilizing, external rational and external irrational. Because such media messages transcend the boundaried between the local communities, they play a destabilizing role in the simulated opinion evolution. At each simulation step, a message is generated randomly, following the probabilities which are the main simulation parameters for the model. These probabilities may, of course, vary in time as the parties’ media strategies change.

The effects of the four kinds of the messages correspond to their description. The mobilizing propaganda tends to strengthen the commitment of the supporters by arousing their agitation. The demobilizing messages (seldom intentional, but quite frequent in reality, due to lack of communication skills) tend to lower the emotional commitment, and, if repeated frequently enough, may turn the agents into neutral non-supporters by ‘boring’ them.

With respect to the propaganda directed outside the party current support base, the rational messages are aimed at convincing non-supporters (neutrals and opponents) to the proposed opinions. The irrational category covers these messages that produce the opposite, backfire effect [98]: they leave the non-supporters with their opinions but in an agitated, hostile state. Quite often the same message may be doubly categorized. For example, it may be a mobilizing one within the current electorate, and, at the same time, irrational outside it—making the non-supporters agitated and more willing to defend their positions through biased processing and the backfire effect. The details of the effects of the messages are described in Table B in S2 File.

Our particular choice of the parameters used to describe the effects of the propaganda is motivated by a desire for an eventual derivation of the model values from the observations of the trends in the media (both traditional and new). The simplicity of the classification, relying on the origin, intended audience and effects (motivating/demotivating, rational/irrational) allows the use of text analyses and datamining techniques. Such analysis of the ‘source’ messages can be combined with a similar analysis of the secondary effects: reactions to these primary messages in the form of comments, tweets, likes/dislikes… These would allow to measure how effective assignment of the type of the strategy, by measuring the alignment of the inferred intention and the observed public reaction. On the one hand, optimistically, if the ‘strengths’ of various types of the propaganda messages derived from the datamining can be matched to the model parameters, then the use of these observed values could be used to forecast the effects on the voting population. Because the political events are largely driven by the communications, such approach could have a better chance than the Google Flu prediction [99–103]. On the other hand, should there be a significant discrepancy, such comparison would allow to improve the basic assumptions of the model. A datamining study of the Polish news landscape and social media is currently planned and awaits a funding decision. Moreover, with respect to the parameters describing the individual reactions, once could recall the remarks of Castellano, Fortunato and Loreto, in their review of the use of physical tools in social analyses [3], addressed to biological models, but equally applicable to the studies of the opinion dynamics: *Quantitative predictions based on this model are difficult because the parameters that serve as input are hard to obtain from actual [] systems*.. Fortunately, some recent experimental works have resulted in quantitative indications of the relative role of the various individual opinion modification factors that may prove to be useful in improving the sociophysical modeling [104, 105].

The combination of the individual opinion and emotion dynamics and the message based communication algorithm has been already used to describe, in a quantitative way, the properties of a discussion forum, including the ratios of expressed emotions, opinions, types of messages (agreements, disagreements, trolling etc.) [39]. The details of the flow of the simulation for each communication event are presented in S4 File.

## 4 Specific simulation conditions

In the current approach we aimed at qualitative description of the real political situation. For this reason, in the following discussion, we will be using the names of the actual parties and political movements, rather than rely on abstract notation. This should facilitate the comparison between the model and the political situation described in Section 2, especially for non-Polish readers. It should be noted that the use of this shorthand notation does not mean either support or lack of support on the part of the author for any of the parties mentioned.

The two-opinion model used in the previous works may be easily extended to a larger number of exclusive opinions. In our case, considering three parties (two major contenders and a newcomer), instead of the seven states corresponding to the two party situation, there are 10 states: one global neutral and three states for each party. A graphical representation of the three party states is included in Fig A in S3 File. However, all the basic rules of the individual opinion dynamics remain the same, as in each event (contact between two agents or an agent responding to a media message) the dynamics remains limited to just two possible alternatives.

To describe any social situation using the Emotion/Information/Opinion model, one has to combine the individual dynamics with a model of the interactions between the involved agents. The communication processes may differ vastly in such aspects as the communication mode (one-to-one, one-to-many), topology of the social network, and the time dependence (e.g. burstiness of the communications). Obviously, an attempt to describe in an accurate way all the intricacies of a political process involving a whole country is beyond our capacities. In the case of the political discussions on the Internet fora [39] we were able to detect the topology of the communications network and to use it in the modeling process.

**Fig 5:**
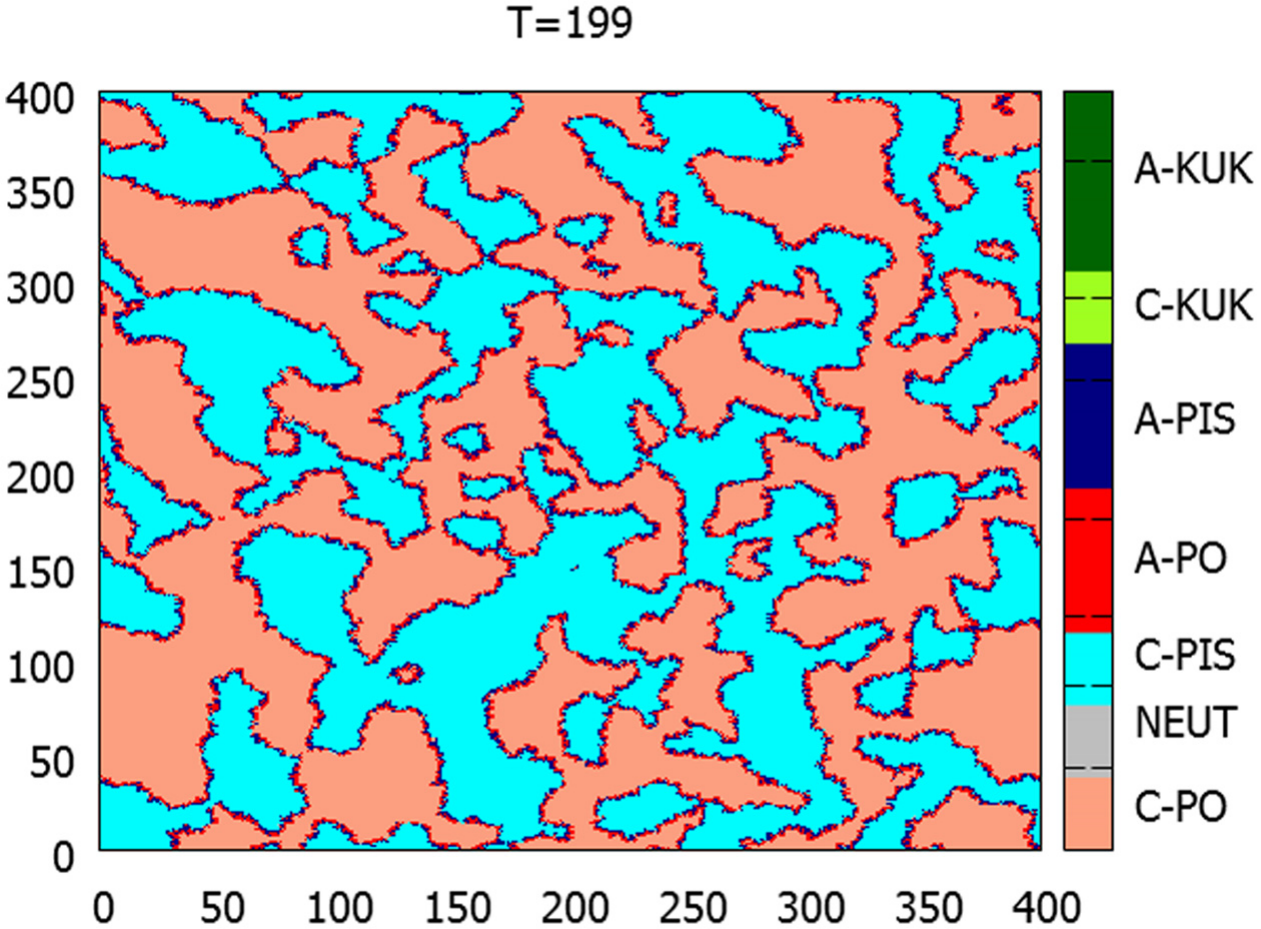
An example of a microscopic snapshot of the system configuration for a random distribution of the initial seed agents. The simulation time is *T* = 199—just before the PO and PIS propaganda messages are switched on. The time corresponds to the evolved starting conditions (*T*_1_). Each point corresponds to a single agent. Light blue points: calm PiS supporters; dark blue: agitated PiS supporters; orange: calm PO supporters; red: agitated PO supporters. Agitated agents are localized at the boundaries of the ‘party held’ domains.

Because the details of the communications processes in the case of a electoral decisions are unknown, and may differ from person to person, we have decided to use a radically simplified option of using a simple 2D square geometry. Such choice has a long history in the ABM literature, to recall here the classic works of Schelling [106] on social segregation or Nowak, Szamrej and Latané [23] on opinion changes. The general properties of the E/I/O model in such 2D geometry have been described in detail in [38]. This choice of a short-range, 2D topology of the interactions is justified, to a certain extent, by the existence of strongly local political preference patterns, as shown in Fig 2. Moreover, the Polish society is characterized by a relatively low mobility, so that within the considered timeframe, the assumption that the local neighborhood of an agent remains the same is a reasonable one. Moreover, the use of 2D geometry (as opposed to more complex social network topologies) allows us to visualize the evolution of the opinions. The system starts in a neutral state, with a small, randomly placed admixture of ‘seed’ agents representing the two parties: PO and PiS. This admixture is at the level of ∼1%. The third party is absent at this stage. We have allowed only short range interactions between the agents, in the Moore neighborhood. Without the media messages, the system evolves to stable domains of shared opinions. Within these domains, agents are surrounded by neighbors sharing the same opinion, so their emotional state becomes calm. Agitation occurs at the boundaries between these domains. Such situation may be seen in Fig 5. This stage of the simulation ends at the time *T*_1_, and may be considered a system preparation phase, in which the social division is created, so that the state at *T*_1_ is a ‘true’ starting condition. In the presented simulations *T*_1_ = 200 Monte Carlo steps per agent, which allows the domains to fully stabilize.

Small modifications of the initial number of seeds representing PO and PiS lead to different ratios of supporters of the two parties at *T*_1_. Up to this moment the system dynamics is fully symmetric between the two parties, and the final configuration at *T*_1_ depends on the initial seed ratios and positions.

**Fig 6:**
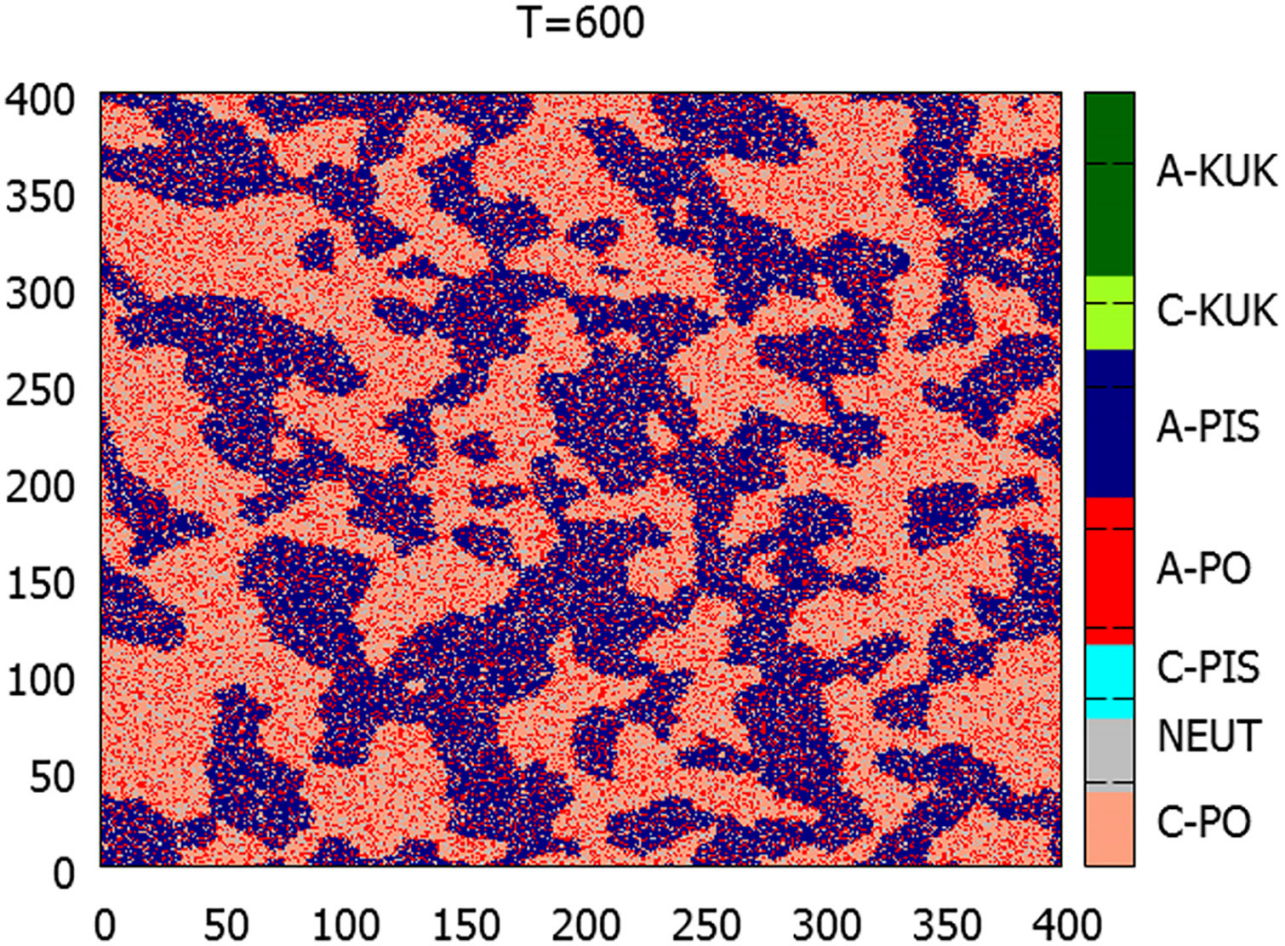
Microscopic snapshot of the system configuration (for the same initial conditions) at *T* = 600—when the effects of the PO and PiS propaganda are well set in. Almost all PiS agents are agitated, while PO domains remain relatively calm.

At the beginning of the ‘true’ simulation, *T*_1_, both major parties ‘switch on’ their propaganda machines. This is where the asymmetry is introduced. The main form of the PO propaganda is rational, with some admixture of demobilizing effects. For PiS, the main effort is internal mobilization, with some ‘spillover’ irrational external effects. The parameters are listed in column 1 in Table 1. Until *T*_2_ = 800 MC steps per agent, the political status quo is reproduced, with most of the PO supporters within their domains remaining calm, while most of the PiS supporters within their domains are turned into the agitated state. A typical snapshot of such situation is shown in Fig 6. The time between *T*1 and *T*2 is denoted as period A.

**Table 1:** Parameters used in the simulations presented in Fig 10. For time period C we provide an average value and 2*σ* range of the parameter sets used to study possible variants of parties’ communication strategies between July and October 2015. The set of values for time period C was chosen by the author on the basis of the observations of party media campaigns before the end of August 2015 and were used to predict the election outcome two months in advance [107].

	Time period A	Time period B	Time period C
MC steps	200–800	800–950	950–1180
Corresponding real time	before March 14	March 14—June 10	June 10—October 30
Message type	Message ratio		
PO mobilizing	0.00	0.00	0.173 ± 0.014
PO demobilizing	0.10	0.05	0.017 ± 0.024
PO rational	0.25	0.15	0.108 ± 0.030
PO irrational	0.00	0.00	0.00
**PO total**	**0.35**	**0.20**	**0.30**
PiS mobilizing	0.40	0.30	0.0294 ± 0.024
PiS demobilizing	0.00	0.00	0.00
PiS rational	0.00	0.01	0.064 ± 0.036
PiS irrational	0.05	0.09	0.042 ± 0.018
**PiS total**	**0.45**	**0.40**	**0.40**
Kukiz mobilizing	0.00	0.10	0.032 ± 0.010
Kukiz demobilizing	0.00	0.00	0.018 ± 0.010
Kukiz rational	0.00	0.06	0.012 ± 0.010
Kukiz irrational	0.00	0.04	0.038 ± 0.010
**Kukiz total**	**0.00**	**0.20**	**0.10**

At *T*_2_ = 800 MC steps the third party enters the political scene, with a mixture of internal and external propaganda messages. This starts the second period (denoted as B in Table 1). *T*_2_ is meant to represent the moment when Mr Kukiz starts to receive media attention in the presidential campaign. The abstract time *T*_2_ = 800 MC was thus set to correspond to March 14, 2015. In the three party setting an agent supporting one of the parties treats messages from both other parties as external, on an equal footing.

**Fig 7:**
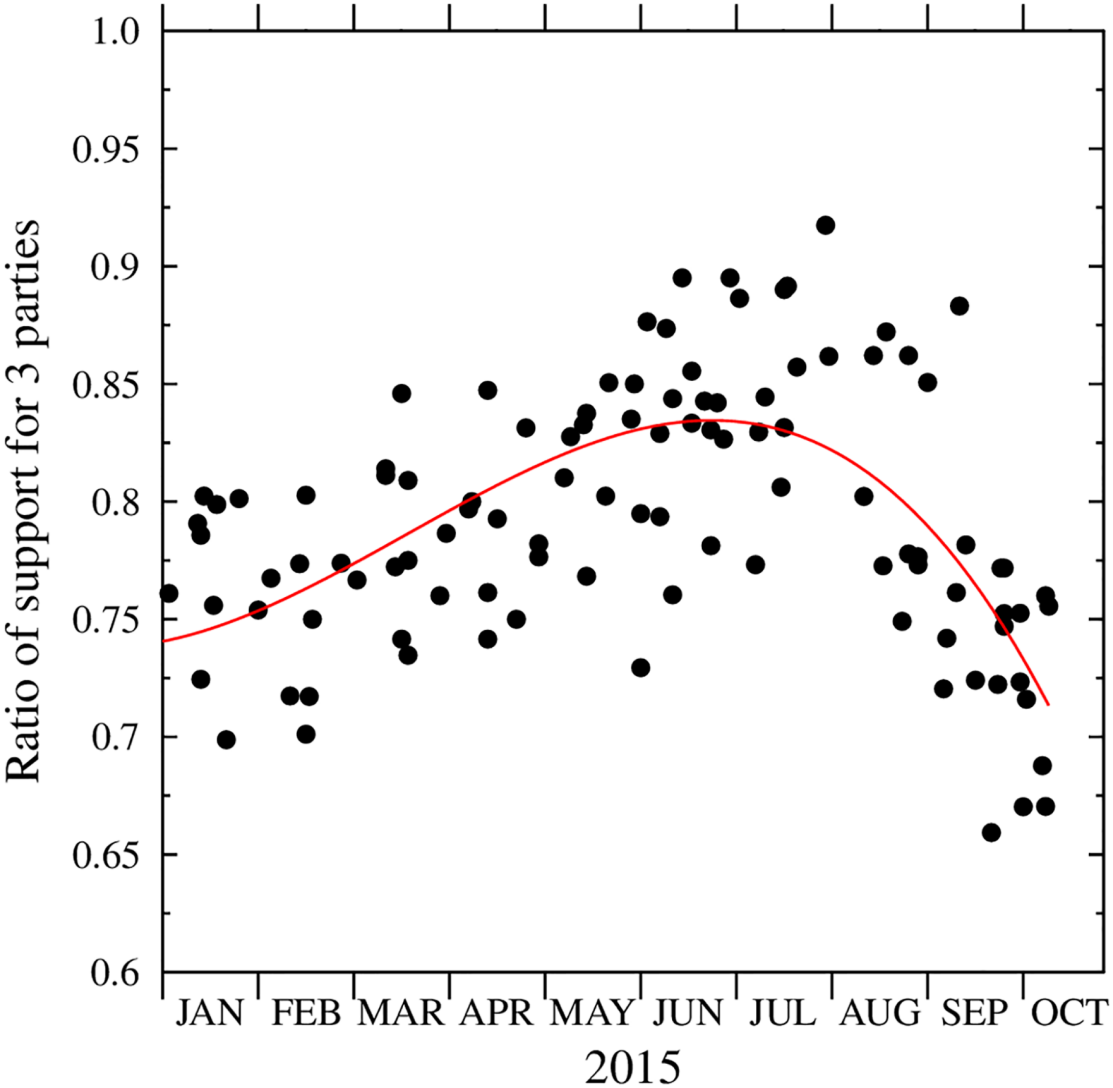
The ratio of the summed support for PiS (together with SP and PJN), PO and Kukiz, in relation to the total reported support for all parties considered within each poll. This allows to normalize the results of the three party agent based model to the real world. Black dots: results for the individual polls, red line: best fit with a third degree polynomial, used in normalizing the raw simulation results.

We note here that to limit the number of the parameters, we have restricted our model to three parties. Within the model, the support for the three parties and the neutral agents always adds up to 100%. To map the results of the three party model onto the real world, one has to ‘normalize’ the simulation results to match the total popularity of the three parties in the poll results. Fortunately, for the period 2015, the observed ratio of the summed popularity of the three parties has followed a rather clear pattern, first increasing and latter dropping sharply. It could be approximated by a third degree polynomial, as shown in Fig 7. This allowed us to normalize the simulation results to the reported poll results, by multiplying the raw numbers (summing to 100%) by the value of the smoothed three-party support in the actual polls. This normalization, which is independent of the simulation parameters has allowed to reproduce the observed support quite well.

## 5 Model results

### 5.1 Breakdown of duopoly: presidential elections (May 2015)

The approach used in the current paper is twofold. The first part was to determine the model parameters (especially the parameters describing the party propaganda strategies) which lead to a good agreement with the real world data (up to the presidential elections), and, at the same time, remain in a reasonable agreement with the estimations of the actual propaganda intensities.

The second part is to study the effects of possible future strategies, chosen by the parties and to predict the results of the parliamentary elections scheduled for late October. This will be discussed in the next section.

The parameters used in the model are designed to correspond to some features of reality, so that, in principle, they could be compared with certain characteristics of the social system (such as the relative frequencies of media messages of the four types) or of the individual perception and response. Unfortunately, in this quick communication, there is no such comparison, backed by objective data. The ranges of the parameters are instead based in the author’s estimates of the actual social activity in Poland.

In the estimation of the propaganda parameters we note that the appearance of Mr Kukiz as ‘news’ naturally decreased the attention given to the two other parties. Moreover, to reflect a relatively lackluster campaign of the PO presidential candidate, the activity of PO was assumed to drop much more than that of PiS. PO messages remained split between internal demobilizing and external rational, while PiS largely preserved the focus on internal mobilizing and external irrational. As the campaign entered later stages, at least some of the PiS propaganda became addressed outside the core support base in a rational way, reflected by a small value of the corresponding parameter. The media communications related to Mr Kukiz (assumed to reach 20% of the media stream) were split between mobilization of the newly won supporters (10%), some (6%) rational messages, designed to win new supporters and a smaller fraction (4%) of messages perceived as irrational. Again, these values are reported in Table 1.

The range of values of the above parameters for the time periods A and B was selected by the author as representative of the overall media strategies by the three concerned parties. The specific values reported in Table 1 are then the results of an optimization of the simulations agreement with the poll results for the two periods. Due to the presence of rather significant errors in the polling process, the fitting process does not lead to unique values of the parameters within the range, thus the values should be treated as representative ones.

This phase of the simulations is assumed to end at *T*_3_ = 950 MC steps, which we set to correspond to June 10, at which time the results of the presidential elections won by the PiS candidate were absorbed by the population and Mr Kukiz begun in earnest to form his political movement.

**Fig 8:**
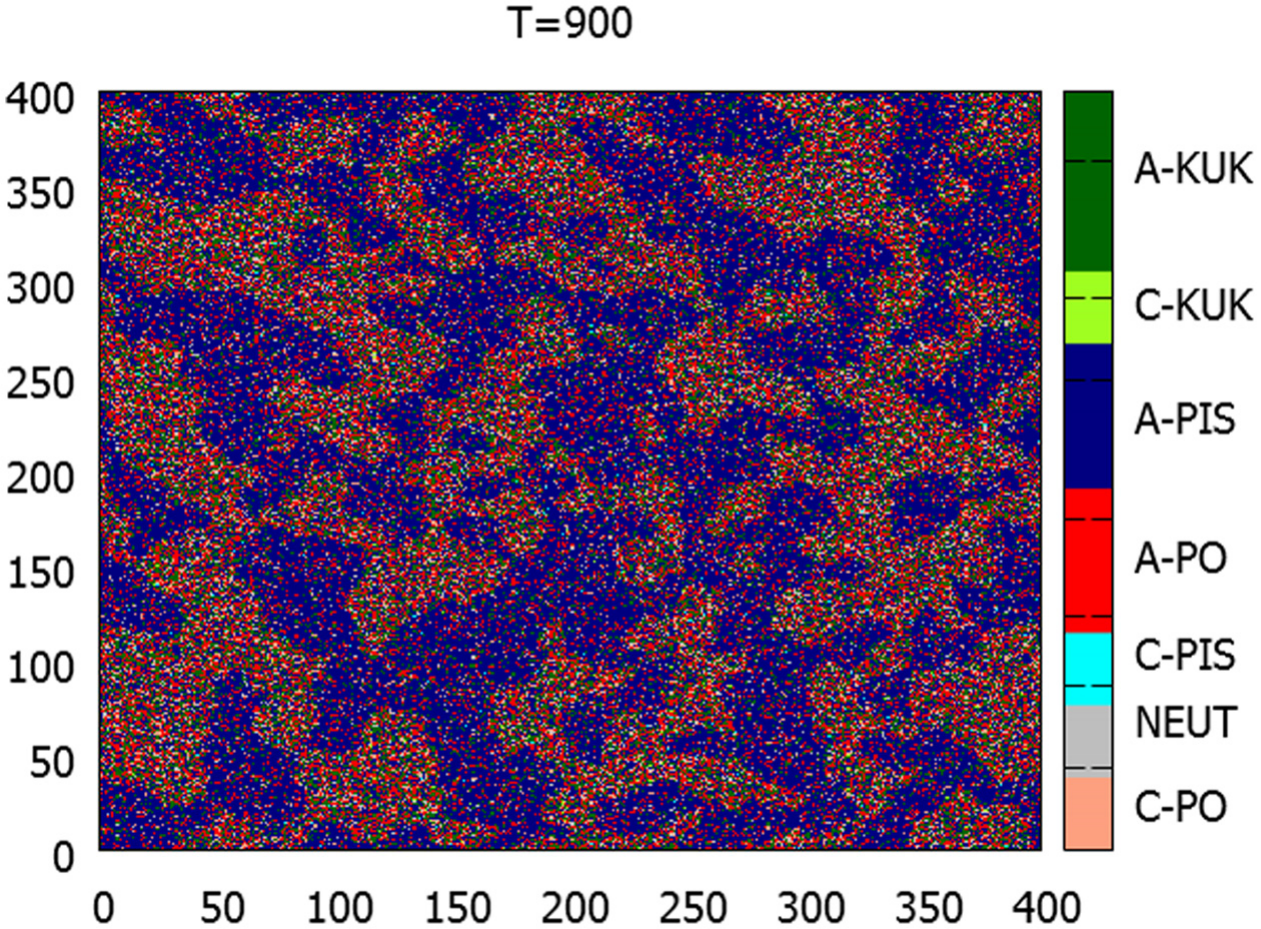
Microscopic snapshot of the system configuration at *T* = 900—one hundred time steps after Kukiz propaganda started. The agitated PiS agents are largely immune to it, while the previously calm PO domains become invaded by Kukiz supporters. Light green: calm Kukiz supporters; dark green: agitated Kukiz supporters.

The appearance of Mr Kukiz affected mostly the calm supporters of PO. This can be seen in Fig 8, where the admixture of the green points, representing the Kukiz supporters occurs mostly within the domains previously occupied by PO voters.

**Fig 9:**
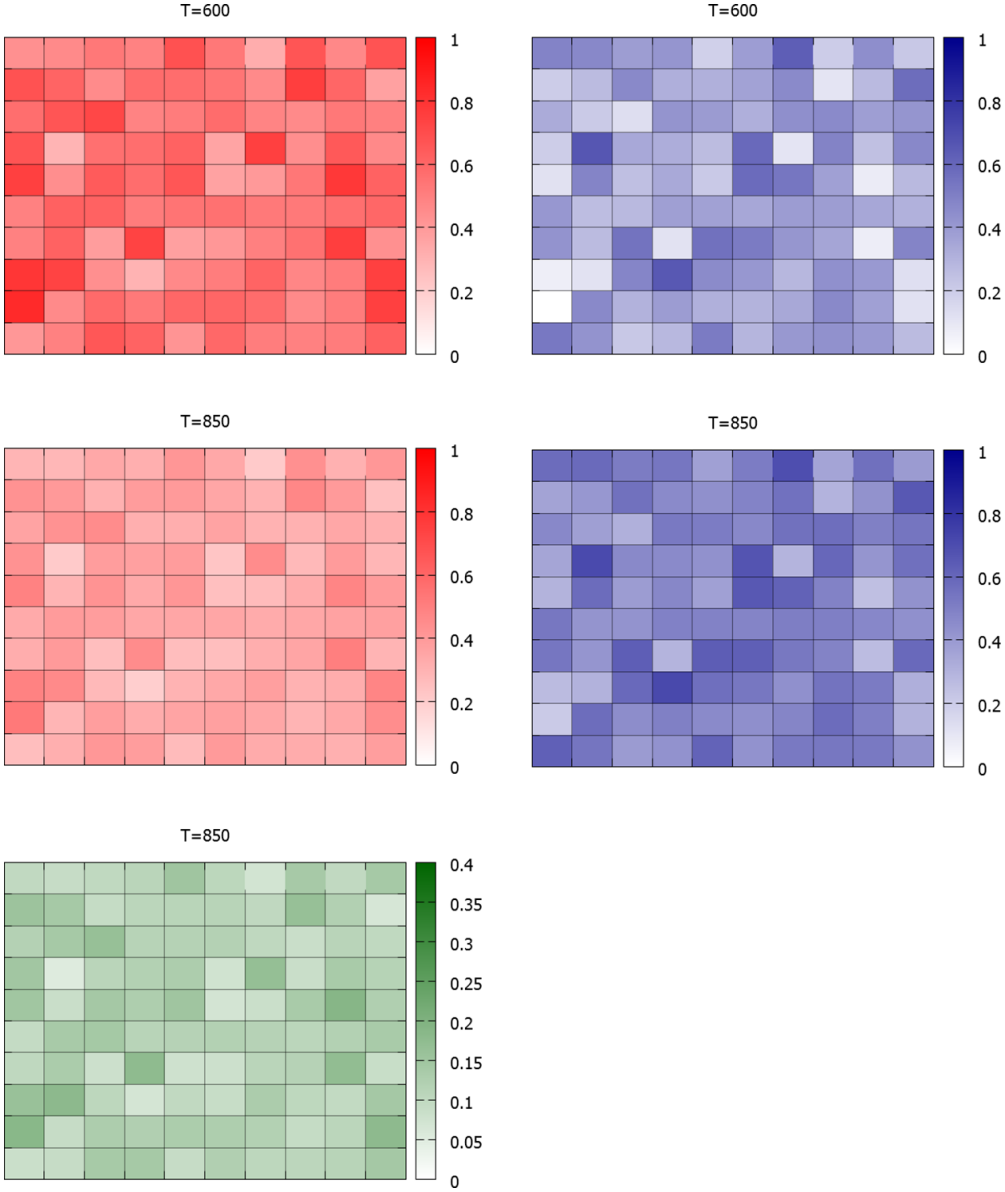
Macroscopic evolution of the support for PO, PiS and Kukiz in the model. 40 × 40 squares form ‘constituencies’ and the panels show the changes in the averaged support for these constituencies. Red: PO, blue: PiS, green: Kukiz Top row: simulation time *T* = 600—before Kukiz party appears, middle and bottom rows: *T* = 850, 50 timesteps after Kukiz propaganda starts. The model reproduces qualitatively the observations from Fig 2, namely the largest gains of the newcomer (Kukiz) party are in the locations where the party employing the calm, rational communication strategy (PO) was strongest. Kukiz gains in regions dominated by highly agitated PiS supporters are the weakest. moreover, the general weakening of PO support increases the PiS lead in the constituencies previously dominated by it.

This is even more clear in Fig 9, where the individual agents were grouped into ‘constituencies’, made of 20 × 20 squares. The relative numbers of the supporters of the three parties in each constituency are depicted by the intensity of color. The figure compares the relative support at *T* = 600 (when Kukiz has not yet appeared) and at *T* = 850 (when its support has already grown significantly). While the support for PiS (blue) increases only weakly, the support for PO falls significantly (red). Moreover, the growth of the Kukiz supporters (green) is strongest exactly in the ‘strongholds’ of PO. These observations may be compared with the real data in Fig 2: the model is qualitatively similar to the reality.

**Fig 10:**
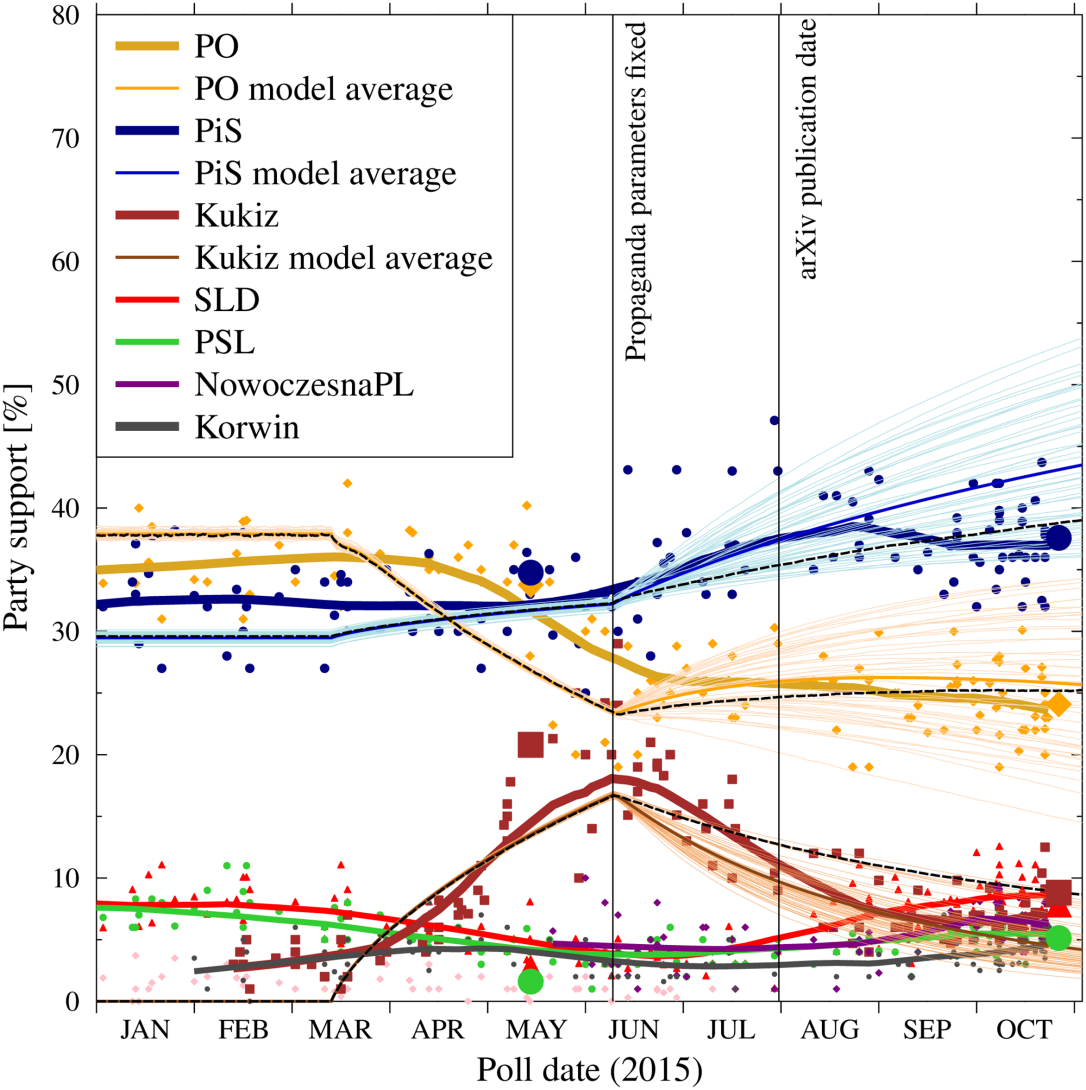
Comparison of the model predictions with the actual evolution of the support for major political parties in 2015. The vertical lines denote the fixing of the individual simulation parameters for time period C and the publication date of the arXiv preprint [107]. The points and thick lines are the actual poll results and their averages, the same as in Fig 4. The light, thin lines are individual simulation results for PO, PiS and Kukiz, calculated using the set of values for time period C was chosen by the author on the basis of the observations of party media campaigns before the end of August 2015 and presented in Table 1. The darker thin lines are ensemble averages for these simulations. Large symbols indicate the results of the presidential (May) and parliamentary (October) elections. The black dashed lines are the results of the simulations for a specific set of parameters listed in Table 2.

These similarities are accompanied by a quantitative agreement of the model data on the normalized popularity of the three parties shown in Fig 10. The model reproduces the rapid growth of the popularity of Mr Kukiz at the expense of PO in the period of March to May 2015, with only slight change (upwards) of PiS results.

### 5.2 Prediction of the results of the parliamentary elections (October 2015)

Encouraged by the quantitative agreement of the model and the data on polls and results of the presidential elections, we have attempted to use the model to predict the possible range of results of the forthcoming parliamentary elections. During June and July 2015, we have run multiple simulations, using the initial set of parameters defined for the time periods A and B, but with an ensemble of slightly different propaganda parameters, representing various possible strategies of the three parties in the starting parliamentary campaign. These parameters covered the last time period of the simulations, from *T* = 950 MC steps (June 10th) till *T* = 1185 MC steps (October 25th), denoted as period C. The general choice of the parameters for this period is based on the observed significant changes in the actual media strategies of the parties, visible in June and July. At the moment these simulations were run, the choice of the actual persuasion tactics and overall media coverage of the parties could, of course, only be guessed, so we have assumed an unchanged strategy for the whole pre-election period. The parameters were therefore assumed to remain constant till the election date.

Let us start with Mr Kukiz: after the initially high media coverage during the presidential campaign and shortly after, the stream of news has dropped sharply. Moreover, as we have noted, Mr Kukiz begun, almost immediately, to quarrel with some of his backers, and to send out very mixed signals, due to the diversity of the candidates grouping around him. To reflect this we have decreased his part of the media messages to 10%, out of which roughly half were internal and half external. A part of the internal messages continued too mobilize, but a significant fraction should be considered demobilizing. Also the fraction of messages externally perceived as irrational has increased sharply, with respect to the total ratio of messages related to Mr Kukiz and his movement.

In contrast, the PiS strategy has changed only slightly. As before, most of the messages are still addressed to the current electoral base with mobilizing effects. But now the majority of the external messages (addressed to the supporters of PO and Mr Kukiz and to the undecided) is now rational and calm, intended to win new supporters. The politicians leading the campaign were chosen based on their conciliatory, calm, rational and positive public image. For this reason, Ms Szydło was chosen as the PM designate and not the party leader, Mr Kaczyński. Most of the controversial, loaded topics, so prominently visible in the past, were absent from the pubic presentations or downplayed. This is reflected, in our simulations, by a much larger probability of external rational messages. As it turned out, in August—September, this ‘rational’ strategy has resulted in a somewhat ‘boring’ image of Ms Szydło, who was perceived as repeating the same messages. Noting this, PiS has, in the latest stages of the campaign, re-introduced the more controversial topic and persons. This action has not been included as a separate change in the model parameters.

The largest change could be observed in the PO strategy: woken up by the loss of the presidency, the party increased its activity. While still large part of the effort was directed externally using rational appeals, internal communication exhibited a strong shift towards mobilizing messages (based mostly on the negative emotions connected with ‘what would happen should PiS win’).

For the fourth simulation period (C) we have run 50 simulations with slightly different values of the media parameters. The last column in Table 1 presents the average values together with the 2*σ* range of the variation between the simulation runs. The evolution of the support for each of such runs is shown as thin lines in Fig 10.

We remind here that for each simulation, a single set of parameters remained fixed for the entire period between June 10th and October 25th. Thus, our model does not include any fundamental shifts of the strategies (which were rather minor in reality), nor any one-time events such as highly publicized speeches or debates. Still, despite these shortcomings, the predicted range of the evolving party popularity has remained in reasonable agreement with the observed values. An early version of the current work has been published on the arXiv server [107], with the goal of recording predictions before the actual elections occur.

The predicted election results, based on the propaganda estimates listed in Table 1 and the base model dynamics described in Section 3, were (the average of the ensemble of simulations and the ± width of the results distribution): PO $26_{-8}^{+8}$ percentage points; PiS $43_{-8}^{+10}$ percentage points; Kukiz’15 $5_{-2}^{+4}$ percentage points. As our model considered only these three parties, the results for the others were entered only via the normalization factor, indicating that about 30% of the votes would go to other parties.

The actual results of the elections were: PiS 37.58% of the votes, PO 24.08%, Kukiz’15 8.81%. The parties and coalitions that were not covered in the agent based model have received: SLD coalition 7.55% (below the required coalition threshold), PSL 5.13%,. Nowoczesna 7.60% (both above threshold) KORWiN 4.76%, RAZEM 3.62% (both below party threshold). The ratio of the votes for the three parties used in the simulations was 70.47%, in perfect agreement with the assumed normalization factor used in the model.

The results of the three considered parties fall within the envelopes of the model predictions for the assumed ensemble of the parameters. Moreover, there are combinations of the propaganda parameters that lead to results close to the final voting results. An example of such parameter set for the time period C is shown in Table 2, and the resulting support evolution is shown as dashed black lines in Fig 10.

**Table 2:** An example of the specific values for the parameters for the time period C allowing to reproduce the results of the parliamentary elections. The corresponding evolution of support for the three parties is shown by the dashed black lines in Fig 10.

	Time period C
MC steps	950–1180
Corresponding real time	June 10—October 30
Message type	Message ratio
PO mobilizing	0.16
PO demobilizing	0.019
PO rational	0.121
PO irrational	0.00
**PO total**	**0.30**
PiS mobilizing	0.265
PiS demobilizing	0.005
PiS rational	0.08
PiS irrational	0.05
**PiS total**	**0.40**
Kukiz mobilizing	0.038
Kukiz demobilizing	0.012
Kukiz rational	0.03
Kukiz irrational	0.02
**Kukiz total**	**0.10**

## 6 Discussion

### 6.1 Model limitations

We are fully aware of severe limitations of our model, as an attempt to describe, even qualitatively, a real social situation.

Firstly, the model does not take into account the appearance of new voters. Bearing in mind that the young voters are influenced to a very large extent by their peers (rather than by their elders), their entry into the electoral system disturbs it in more way than one. An extension of the current model aimed at allowing such dynamical ‘flow’ of voters is planned.

Secondly, the voting preferences depend to a large extent on specific events, such as scandals involving politicians, or even results of football matches. These events typically have short term effects, but their accumulation may shift the balance more permanently. Of course, the model does not contain such events and their effects. Moreover, the assumed asymmetry in the propaganda (only calm messages for PO, only aggressive messages from PiS) does not correspond to reality. In particular, the PO communications strategy contains some admixture of messages promoting fear of what would happen should PiS come into power. These messages, while fewer in number and visibility, were always present, and may serve to strengthen the resolve of the PO electoral base.

Thirdly, the model focuses on just three parties, while the actual situation is much more complex. As may be seen from Fig 1 until the end of 2014 the summed popularity of the ‘minor’ parties at the time (TR, SLD, PSL) exceeded the difference between PO and PiS, so that these parties could aspire to tip the scales in the political situation—a role quite successfully performed by PSL as PO coalition partner. Moreover, the model does not take into account the existence of inflexible supporters for the various parties, assuming that in principle all agents are free to change their sympathies. The introduction of small groups of die-hard supporters would change the quantitative results of the model.

Lastly, we note that despite the benefits of the simplification of the continuous cusp catastrophe model brought by the use of the discrete states, the simulation results depend on the chopice of a relatively large number of parameters. Of special importance are, of course, the initial ratios of the ‘seed’ agents in the preparatory phase, which determine the numbers of supporters at the stability period and the parameters describing the media strategies of the contending parties. Fig 10 and Table 1 illustrate the effects of relatively small variations of the media parameters on the evolution of the party support.

The problem is quite common to many nonlinear, multi-parameter models: even a very good fit between the model results and the empirical observations is not an indicator that the particular set of the input parameters is the unique, ‘true’ value. In our case, the problem is worsened by a rather large error in the observed values (poll results) to which the model should correspond, typically in the 2%–4% range.

For these reasons, the predictive power of the E/I/T model is limited. Still, it has been the intention to construct the it in a way, that the parameters have some meaning in relation to the real world, for example the probabilities of becoming agitated or calmed down by a social encounter or the relative combined frequency and impact of media campaigns. In this way one can, in a limited way, check if the values used in the ‘best fit’ simulations are comparable with the actual situation.

### 6.2 Comparison with the case of Ruch Palikota (2011-2014)

It is quite interesting to note that an additional support for the model may be derived from the fate of the Ruch Palikota/ Twoj Ruch (TR) party, which resembled a lot the fate of Mr Kukiz, although on a smaller scale.

The first sign that the two communication strategies lead to different results appeared in 2011. A new political movement, led by Mr Janusz Palikot, a dissident from PO, and named, not surprisingly, Ruch Palikota, (Palikot’s Movement, later renamed to Twoj Ruch, Your Move) has gained popularity, becoming the third power in the parliament with over 10% of votes. Some later polls have shown its popularity reaching over 20%, as shown in Fig 1, pink dots/lines. As Fig 2 shows, the support for RP came from regions of Poland where PO was dominant. This is not surprising, given that the program of RP was, initially, rather similar to that of PO. The appearance of RP is correlated with a significant drop of declared support for PO, and coincides with the period where PiS achieves a small, but non-negligible lead (Fig 1).

This party was formed before the elections in 2011, by a prominent member of PO, Mr Janusz Palikot. It has very quickly gathered sizable support, as shown in Fig 1. In the 2011 parliamentary elections it became the third power, surpassing both PSL and SLD, and gathering 10.02% of the votes. These votes were mostly from previous supporters of PO. In fact the evolution of PO, PiS and TR support in 2011–2014 strongly resembles the shape shown by the model simulations. TR success is accompanied by the fall in PO popularity and growth in support of PiS. In fact, the geography of support for RP/TR in the 2011 parliamentary elections follows the same pattern as the one mentioned in connection with Mr Kukiz (see Fig 2), that is the highest popularity was achieved in some regions previously dominated by PO, while the gains were the weakest in the regions dominated by PiS. The similarity between the two parties rests largely on their dependence on the strong personalities of their leaders.

After the initial gains, Ruch Palikota popularity begun to decrease gradually, following roughly exponential curve.

It is quite interesting that for both RP/TR and Kukiz party, it is possible to use as the trends the same form of the analytic function *A* exp(−(*T* − *T*_0_)/*τ*_2_)/(1 + exp(−(*T* − *T*_0_)/*τ*_1_), being a combination of the initial logistic growth with the speed dictated by *τ*_1_ and the subsequent exponential decay, with the speed dictated by *τ*_2_. The best fit values of these growth/decay times are, respectively *τ*_1_ = 0.04 year and *τ*_2_ = 0.071 year for Kukiz party (although the latter value is obtained from just a few data points), and *τ*_1_ = 0.081 year and *τ*_2_ = 1.83 year for RP/TR.

Interestingly, the drop in the RP popularity was not accompanied by a return of the supporters to the PO base. The slight upturn of PO rankings in 2013 and 2014, after the RP/TR appeal begun to wane should be rather attributed to the shift in the party political strategy, taking a decidedly left turn and to the personal success of Mr Tusk, in his election to the post of the President of the European Council, a huge propaganda coup for the party. The success was coupled with the shift of PO messages towards the left. Such strategy was possible in 2014 due to a totally chaotic management of the social-democratic party (SLD), which has lost almost 10% points, mostly moving to the PO base.

With respect to the 2015 threat posed to PO by Mr Kukiz movement, we note that the scale of the intrusion was almost two times higher, and that there were no more such ‘reserves’—the minority parties are already reduced to the single figure die-hard core electorates, and thus there is no place left for another policy shift for PO. Moreover, the coincidence of the two elections within 5 months has allowed PiS to effectively capitalize on the PO weakness and to achieve the success in both elections.

### 6.3 Why didn’t PiS achieve the predicted 43%?

Until mid-August, the averaged poll results of PiS and the ensemble average of the ABM (for the range of the parameters chosen in July) were practically indistinguishable. The model average, extended to end of October, predicted that PiS would obtain a 43% of the vote, most likely sufficient for a constitutional majority. Yet since late August the reported support first dropped slightly and then stabilized at 36%–38% range. While the difference is well within the range of the values considered in the parameter ensemble, the divergence of the model and the poll results seems worth looking into.

As we have noted before, if one forgets about the ABM results, it is possible to view the support for PiS in the August-October period as approximately constant. In fact, many political commentators have attributed a drop observed in some polls in September and October (with some results as low as 32%) to the public appearances of Mr Maciarewicz and Mr Kaczyński, during which some inflammatory statements were made and subsequently publicized by the pro-PO media. These appearances have run contrary to the rational and calm campaign, whose face was the official PM designate, Ms Szydło. The conventional explanation for the drop was the negative perception of the aggressive, emotional narrative.

From the viewpoint of our model, the explanation is different. As we noted the two lines begin to diverge much before the events mentioned above. Second, the model mechanisms the drop in the popularity may be attributed to the decreasing efficiency of the calm, rational strategy. Instead of winning over the decreasing ranks of the Kukiz supporters, more and more agents became ‘bored’ with the rational argumentation, and become susceptible to other propaganda. For example, the propaganda coming from the parties other than the three covered directly by the model. In particular, we note the rapid growth of two more newcomers to the political scene,. Nowoczesna and Razem, to certain extent, the growth of support for SLD, which grew from 3% to more than 7%. These gains together account for over 15% of the changed votes that did not go to any of the parties considered in the model. The lack of the success of PiS in locking in the PO deserters (via their brief flirt with Kukiz), may have resulted in the kink in the PiS poll results and the subsequent results below the model prediction.

The hypothesis outlined above may be compared with the detailed studies of the flows of electorate for the 2015 elections, once the data become available. The exit-poll results gathered by IPSOS on 25 October 2015 (poll commissioned jointly by Telewizja Polska, TVN 24 and Polsat News) confirm the stability of the PiS electoral base (89.7% of 2011 supporters remained faithful in 2015). On the other hand, almost 48% of voters who have supported PO in 2011 have drifted to other parties (most notably to. Nowoczesna—13.3%, PiS—10.9%, Kukiz’15—6.4% and SLD—6.3%). There are no data (as yet) on the 12% of the voters who have supported Mr Kukiz in May, but failed to vote for him in October.

### 6.4 General applicability of the model

Despite the problems mentioned above, the model, through a simple action of taking into account the asymmetry of communication strategies of the two dominant parties, reproduces the recent shift in the Polish politics surprisingly well. In fact, the success of the model may be due to the fact that the initial conditions in Poland were ‘prepared’ in a special (perhaps unique) way. The long term asymmetry of the PO and PiS media strategies, coupled with the approximately equal size of the voter base, has allowed to use simplified initial conditions for the agent based model, without using too many parameters.

On the other hand, the Emotion/Information/Opinion model may be applied to other circumstances, allowing us to speculate on political environments beyond Poland.

We note first, that strong political polarization is observed in many countries, the leading example being the US. Today it seems a permanent feature of the political system, leading to increased levels of misunderstanding, conflict and even violence. These phenomena have been extensively studied with respect to the US political preferences (see, for example, [55, 108, 109]). In our model, the stability of the polarized bipartisan scenario results from the highly emotional nature of interpersonal contacts and party propaganda strategies. This allows the parties to protect their core support bases, but it has a definite drawback: practically all voters in the system are in agitated states, unable do rationally process any arguments contrary to their current beliefs. Depending on the value at which the division stabilizes, the system may experience drastic swings in policies (if the ratio is close to 50/50, as in the US case) when one side temporarily dominates, or clinch and indecision (if various levels of the governing structure fall into the opposing camps, creating a hostile cohabitation situation).

The current model can also be applied to situations where there is a stronger initial preference asymmetry. In such a case, the relevance of the ‘internal competition’ in keeping up the high emotional commitment of the voters is much more difficult. The obvious strategy is then to introduce the external ‘mobilizing’ elements, such as an enemy in a war-like scenario. One could mention here the unprecedented level of support that Mr Putin enjoys in Russia, despite the negative economic and social effects of the sanctions due to the involvement in the Ukrainian crisis, Crimea annexation and the costs of the military participation in the Syrian/ISIS war, with the popular approval rate of over 89% in October 2015 (Source: http://edition.cnn.com/2015/10/22/europe/russia-putin-poll/).

Unfortunately, both the observations of the real-world politics and the model indicate that the negative effects of the dominance of emotions over rational thinking and impartial considerations are strongly embedded in the democratic systems. The mitigating approaches based on providing the voter with better information are largely naive, as they do not take into account the phenomena of emotional biased processing, agenda setting, motivated reasoning, selective exposure and perception—to mention just a few social mechanisms driving the processes of formation of the individual and social opinions.

## Supporting Information

S1 File Discretized cusp catastrophe description. (PDF)

S2 File Individual opinion dynamics. (PDF)

S3 File Extension of the binary interactions to three parties. (PDF)

S4 File Simulation process flow. (PDF)
