# Supplementary material for: Quantitative Agent Based Model of Opinion Dynamics: Polish Elections of 2015
Source: PLoS One. 2016 May 12;11(5):e0155098. doi: 10.1371/journal.pone.0155098 (PMC4865045; doi:10.1371/journal.pone.0155098)
Supplement: S1 File — (PDF) [file pone.0155098.s001.pdf]

S1 file – Discretized cusp catastrophe description

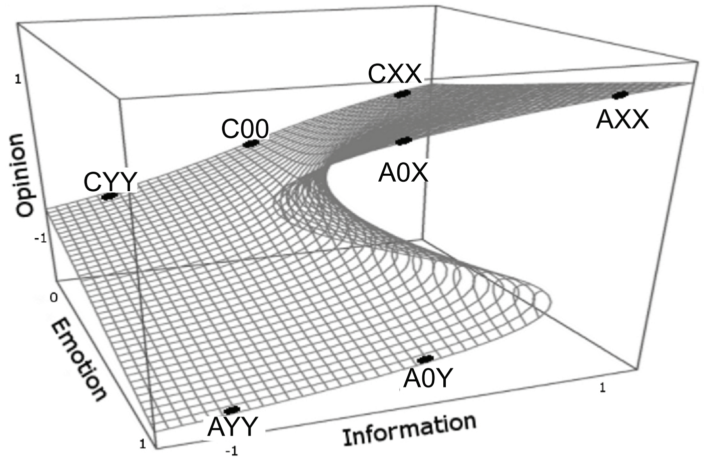

Figure A: Graphical representation of the discretized version of the cusp catastrophe model of the individual opinion dynamics for the simplest case of two possible opinions, X and Y. Instead of the continuum of states described by values of information and emotional involvement, the approach focuses on only a few states. These are characterized by the agent emotional state (calm C or agitated A), information about the issue (favoring X, favoring Y or no information 0) and opinion (favoring X, favoring Y or no information 0). Thus the states may be denoted by simple acronyms, e.g. AXX. There are seven independent agent states in the case of two competing opinions.

The simulations are based on a simplified, discrete version of the cusp catastrophe model of opinion change, with the information on the issue in question and the emotional involvement as the two variables. The discretization of the model means that instead of the two-dimensional continuum of the states we choose only a few points, representing the simplified agent states (Figure A). These discrete agent states correspond to combinations of the agent emotional involvement (calm C or agitated A) and the information about the issue in question and the resulting opinion.
