# Supplementary material for: Quantitative Agent Based Model of Opinion Dynamics: Polish Elections of 2015
Source: PLoS One. 2016 May 12;11(5):e0155098. doi: 10.1371/journal.pone.0155098 (PMC4865045; doi:10.1371/journal.pone.0155098)
Supplement: S2 File — (PDF) [file pone.0155098.s002.pdf]

# S2 file – Individual opinion dynamics

The discretization of the cusp catastrophe model allows to use a much simpler dynamics of the individual agent responses to encounters with other agents or reactions to mass media. These can be described by a finite transition matrix, combining the initial agent state and the characteristics of the agent it interacts with, or of the media messages. These are presented in Tables I and II. In most cases the changes in the agent state (emotion, information and opinion) are deterministic. The few exceptions are related to the processes of rising or lowering of the emotional state. An agitation probability $p_a$ describes a situation in which an encounter of a calm agent with a calm agent or a rational message supporting the opposite view, instead of leading to a change of opinion, irritates and angers the agent, so that its opinion remains unchanged, but the emotional state becomes agitated.

Conversely, the calming probability $p_c$ describes a situation, in which and agitated agent can become convinced by a calm opponent.

| | Initial agent state | | | |
|---|---|---|---|---|
| Neighbor state | Calm, uninformed, neutral (C00) | Calm, informed, X supporter (CXX) | Agitated, informed, X supporter (AXX) | Agitated, uninformed, X supporter (A0X) |
| Neutral neighbor (C00) | Unchanged | Unchanged | CXX | CXX |
| Same party neighbors | | | | |
| Calm,informed, X supporter (CXX) | CXX | Unchanged | CXX | CXX |
| Agitated,informed, X supporter (AXX) | Unchanged | Unchanged | Unchanged | Unchanged |
| Agitated, uninformed, X supporter (A0X) | Unchanged | Unchanged | Unchanged | Unchanged |
| Opposing party neighbors | | | | |
| Calm,informed, Y supporter (CYY) | CYY | AXX ($p_a$)<br>C00 ($1-p_a$) | A0X ($1-p_c$)<br>C00 ($p_c$) | Unchanged ($1-p_c$)<br>C00 ($p_c$) |
| Agitated,informed, Y supporter (AYY) | Unchanged | A0X | A0X | Unchanged |
| Agitated, uninformed, Y supporter (A0Y) | Unchanged | AXX | Unchanged | Unchanged |

Table A: Transition matrix between the agent initial states and final ones, depending on the state of the neighbor, with whom the agent is interacting. In general, the interaction with the agents of the same party leaves the agent state unchanged, except when an agitated agent interacts with a calm neighbor, which leads to a calming of the emotional state. Interactions with agents favoring the opposing view are more complex. Calm supporter of party X (CXX), interacting with a calm supporter of party Y (CYY) may become angered (with a probability of $p_a$), or partially convinced (turned into C00). The same agent (CXX), confronted with an agitated supporter of the opposing party (either A0Y or AYY) will turn into an agitated state. For the agitated agents (A0X or AXX) meeting with a calm opponent (CYY) may result in conversion to the neutral state (C00) with a calming probability $p_c$ or leave the agent agitated and supporting the original choice. Generally, the only way to change the opinion is via the neutral state C00.

| | Initial agent state | | | |
|---|---|---|---|---|
| Message origin and type | Calm, uninformed, neutral (C00) | Calm, informed, X supporter (CXX) | Agitated, informed, X supporter (AXX) | Agitated, uninformed, X supporter (A0X) |
| Same party messages | | | | |
| Demobilizing message | | C00 | CXX | CXX |
| Mobilizing message | | AXX | Unchanged | Unchanged |
| Opposing party messages | | | | |
| Rational message favoring Y | CYY | AXX ($p_a$)<br>C00 ($1-p_a$) | Unchanged<br>Unchanged | Unchanged<br>Unchanged |
| Irrational message favoring Y | Unchanged | AXX | Unchanged | Unchanged |

Table B: Transition matrix between the agent initial states and final ones, depending on the type and origin of the media message. The demobilizing messages (coming from the same party as the agent in question) lower down the emotional state, and, for already calm agents, lead to 'boredom' and to the neutral state C00. External irrational messages simply irritate the opponents, while rational ones may either irritate (with probability $p_a$) or convince, leading to a transition of calm agents to a neutral state.
