# Supplementary material for: Quantitative Agent Based Model of Opinion Dynamics: Polish Elections of 2015
Source: PLoS One. 2016 May 12;11(5):e0155098. doi: 10.1371/journal.pone.0155098 (PMC4865045; doi:10.1371/journal.pone.0155098)
Supplement: S3 File — (PDF) [file pone.0155098.s003.pdf]

S3 file – Extension of the binary interactions to three parties

The original cusp catastrophe model, as well as its discretized version, are constructed to correspond to a situation in which two possible opinions are considered. The political arena may offer the voters more choices, so it is reasonable to extend the opinion model beyond the binary limit. The fact that the dynamics of the opinion change relies on discrete events (either encounters between agents or responses to media messages) allows an easy adaptation of the E/I/O model to a three-party (or even multi-party) situation.

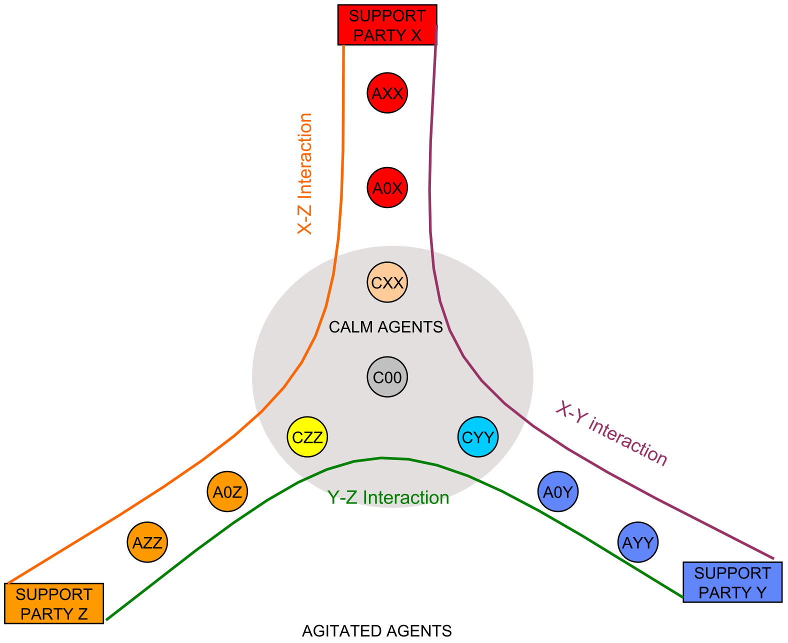

Figure A: Graphical representation of the three-party model. Any communication between a pair of agents will result in the bipartisan dynamics described in Table A in S2 file, and therefore can be described in the seven states of the E/I/O model with the appropriate choice of the two involved parties, for example the case of agent CZZ receiving the message A0Y. The 'true neutral' C00 state is common for all interactions. It is therefore possible to derive a multiple-choice dynamics from the binary system. In the case of the Polish elections the three parties X, Y, X corresponded to PO, PiS and Kukiz. In fact, the E/I/O model may be used for an arbitrary number of parties, as all the interactions between agents and with the media messages involve only two agents.

Fig A illustrates how this may be achieved. In each encounter between two agents we have only two possible opinions, so that the specific result of such encounter is nicely described within the seven-states specific for the particular choice of the parties, as described in Table A in S2 file. This makes the implementation of multiple choice model quite straightforward.

We should note, however, that there are problems associated with the effective application of the multi-opinion model. These are related to the proper handling of the initial conditions. The more numerous are the allowed agent states, the more difficult is to prepare a 'good' initial state. Most of the agent based models ignore this problem, by starting from some randomized distribution of the agents, satisfying some crude set of conditions (e.g. ratios between specific states), but randomized topologically. Such starting conditions may severely distort the simulation outcome – and they do not correspond to most social situations, where strong correlations between opinions and emotions of the socially linked agents are known to exist.

As mentioned in the main text, the Polish political situation may be considered rather special, as the long period of political duopoly and the significant difference in the communication strategies of the two dominating parties have prepared a setup that was relatively easy to reproduce, by using a 'preparatory' phase in the simulations. In contrast, the application of the model to an arbitrary multiparty system would be much more difficult, without a detailed knowledge about the correlations and clustering of the preferences.
