# Supplementary material for: Quantitative Agent Based Model of Opinion Dynamics: Polish Elections of 2015
Source: PLoS One. 2016 May 12;11(5):e0155098. doi: 10.1371/journal.pone.0155098 (PMC4865045; doi:10.1371/journal.pone.0155098)
Supplement: S4 File — (PDF) [file pone.0155098.s004.pdf]

S4 file – Simulation flow

The program flow consists of separate Monte Carlo timesteps, within which the individual agents may either interact with each other or respond to a news item. Each MC timestep corresponds to, on the average, one interaction per agent.

As noted in the main text, the simulation time T is divided into four periods. The initial period ($1 \leq T < T_1 = 200$) corresponds to the seeding of the full 2D agent domain, and is not, in a strict sense, a part of the opinion change simulation. The second period denoted in the main paper as A ($T_1 \leq T < T_2 = 800$) corresponds to the long period of the two-party dominance. The third period, B, between $T_2 = 800$ and $T_3 = 950$ corresponds to the presidential campaign, and the final one, C, between T_3 and $T_4 = 1180$, corresponds to the parliamentary campaign. The parameters describing the media coverage in each of the periods A, B, and Care listed in Table 1 of the main paper.

Within each of the periods, the flow of the simulation for an individual agent event is shown schematically in Fig A.

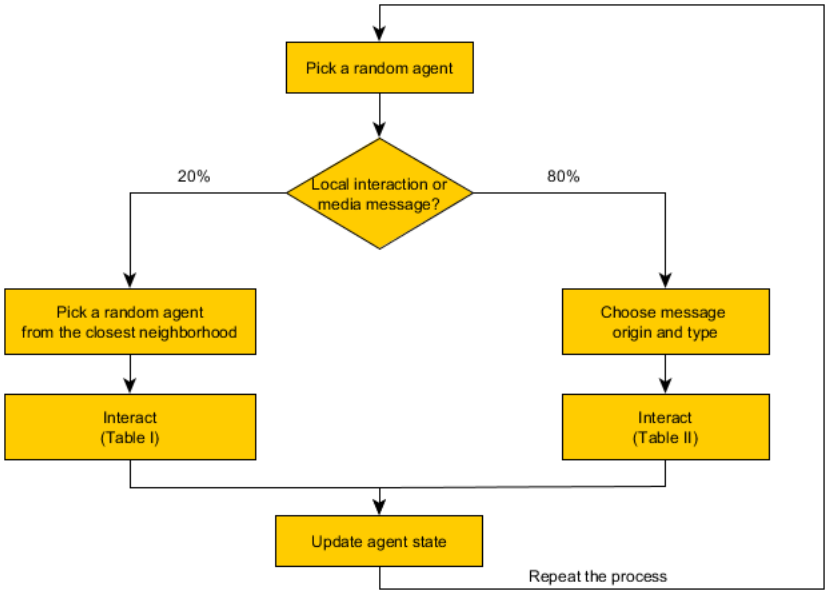

Figure A: Schematic view of the process of adapting the opinions.
